# Supplementary material for: An Approach toward 17-Arylsubstituted Marginatafuran-Type Isospongian Diterpenoids via a Palladium-Catalyzed Heck–Suzuki Cascade Reaction of 16-Bromolambertianic Acid
Source: Molecules. 2022 Apr 20;27(9):2643. doi: 10.3390/molecules27092643 (PMC9102694; doi:10.3390/molecules27092643)

An approach toward 17-arylsubstituted marginatafuran-type isospongian diterpenoids *via* a palladium-catalyzed Heck–Suzuki cascade reaction of 16-bromolambertianic acid

Yurii V. Kharitonov[a] and Elvira E. Shults*[a]

[a]*Laboratory of Medicinal Chemistry, Novosibirsk Institute of Organic Chemistry, Siberian Branch of the Russian Academy of Sciences, Lavrentyev Ave, 9, 630090 Novosibirsk, Russia;*

E-mail: schultz@nioch.nsc.ru

Supporting Information

Figures S1-S46 NMR ^{1}H and ^{13}C spectra of compounds **5**, **7a-j**, **8b,c,d,j**, **9-12**, **13a-c**, **14a**.

(1S,4aR,5S,8aR)-5-(2-(2-bromofuran-3-yl)ethyl)-1,4a-dimethyl-6-methylenedecahydronaphthalene-1-carboxylic acid (**5**) (^{1}H NMR, 400 MHz, $CDCl_3$)

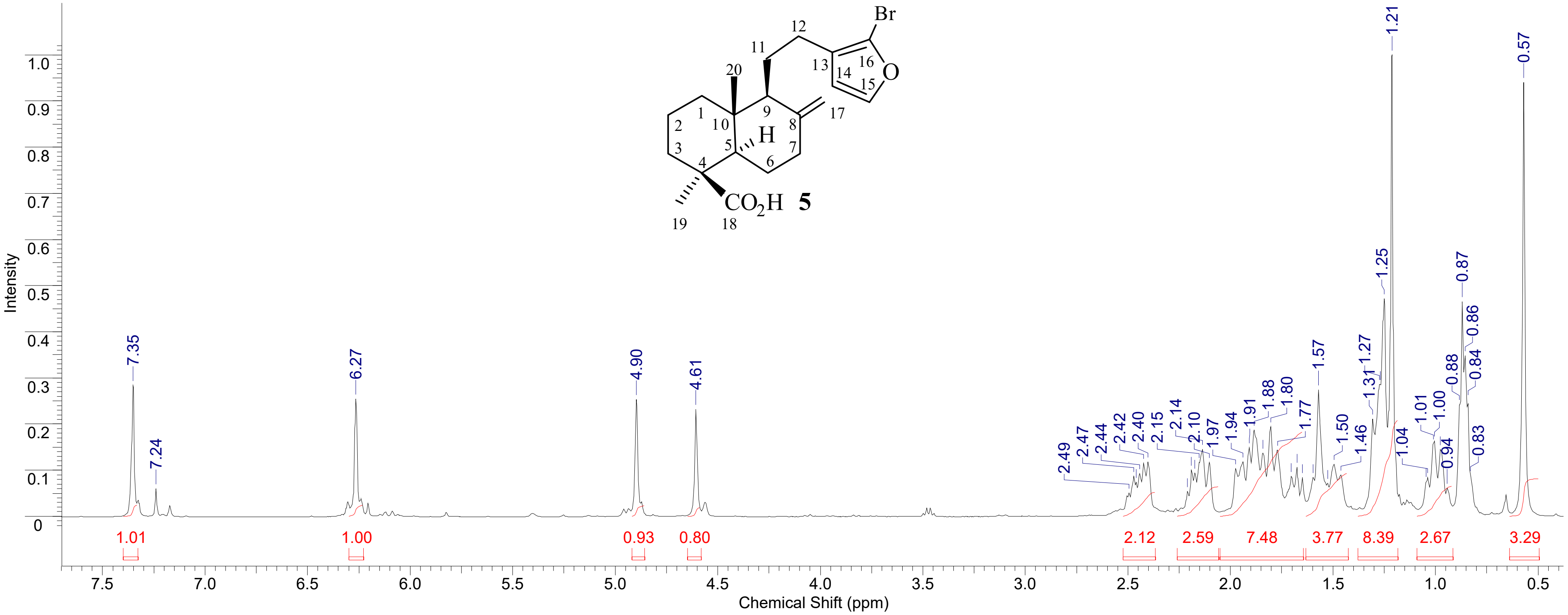

***(1S,4aR,5S,8aR)-5-(2-(2-bromofuran-3-yl)ethyl)-1,4a-dimethyl-6-methylenedecahydronaphthalene-1-carboxylic acid* (5).**

(^{13}C NMR, 126 MHz, $CDCl_3$)

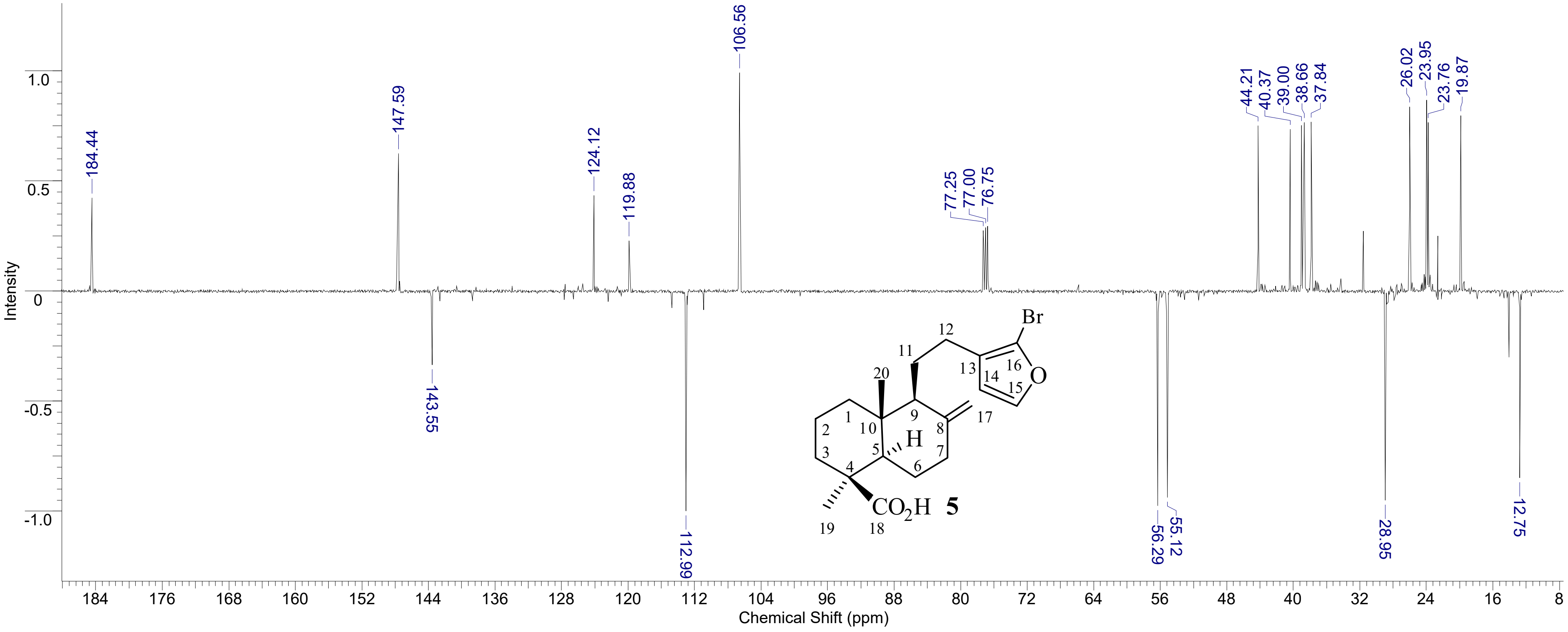

(3bS,5aR,6S,9aR,9bR)-3b-benzyl-6,9a-dimethyl-3b,4,5,5a,6,7,8,9,9a,9b,10,11-dodecahydrophenanthro[1,2-b]furan-6-carboxylic acid (***7a***) (^{1}H NMR, 600 MHz, $CDCl_3$)

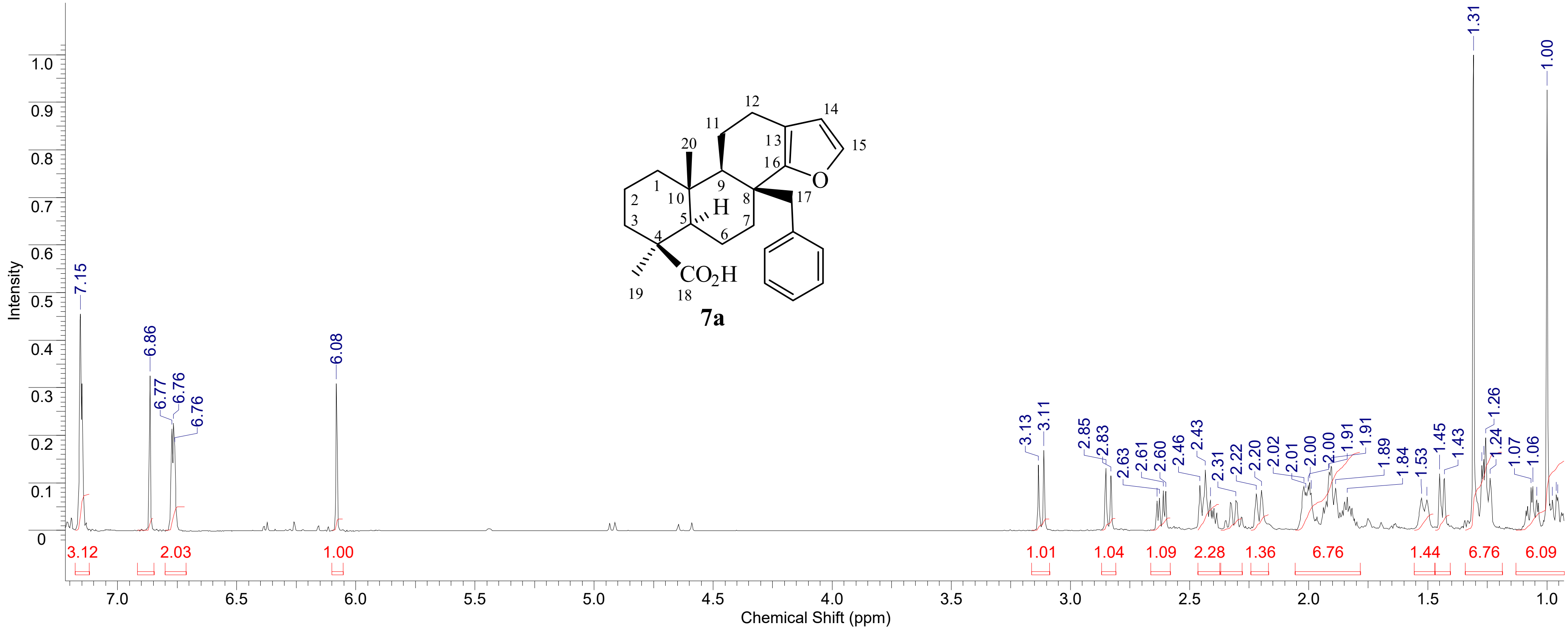

(3bS,5aR,6S,9aR,9bR)-3b-benzyl-6,9a-dimethyl-3b,4,5,5a,6,7,8,9,9a,9b,10,11-dodecahydrophenanthro[1,2-b]furan-6-carboxylic acid (7a)

(^{13}C NMR, 151 MHz, $CDCl_3$)

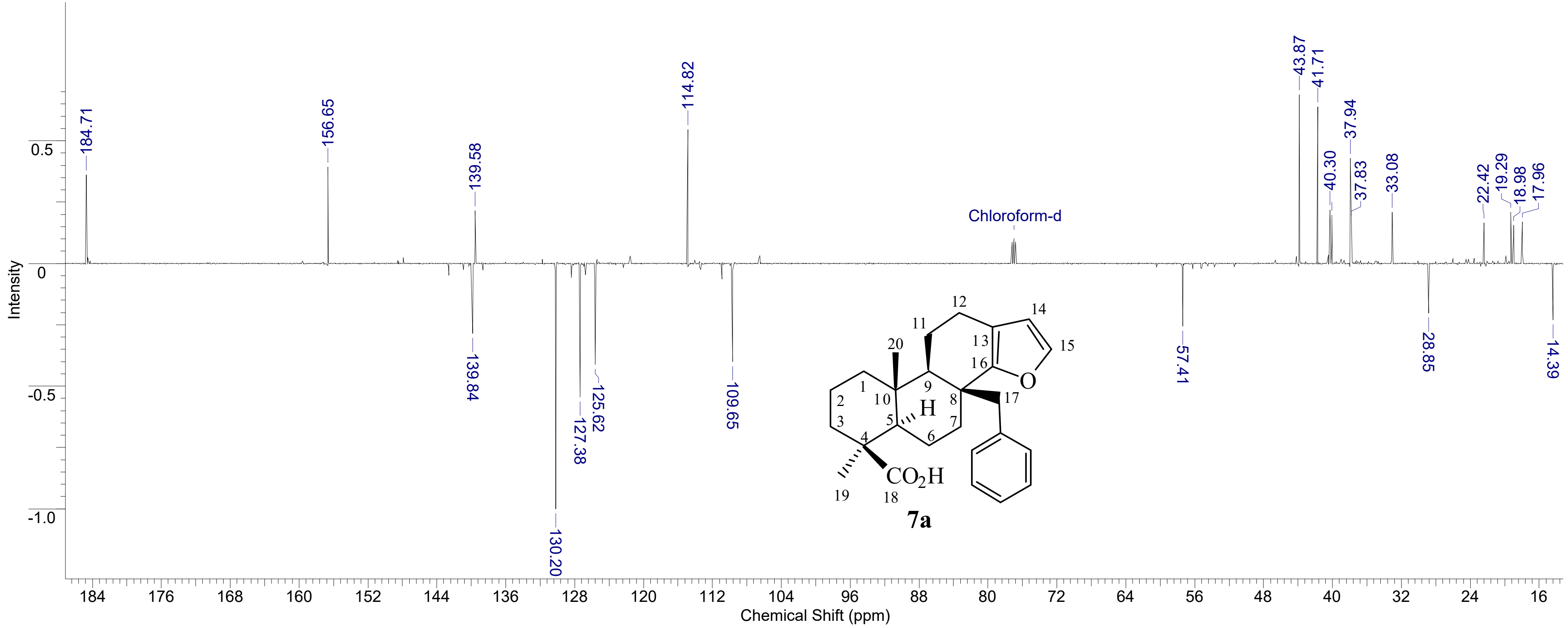

(3bS,5aR,6S,9aR,9bR)-3b-(2-fluorobenzyl)-6,9a-dimethyl-3b,4,5,5a,6,7,8,9,9a,9b,10,11-dodecahydrophenanthro[1,2-b]furan-6-carboxylic acid (**7b**)

(^{1}H NMR, 400 MHz, $CDCl_3$)

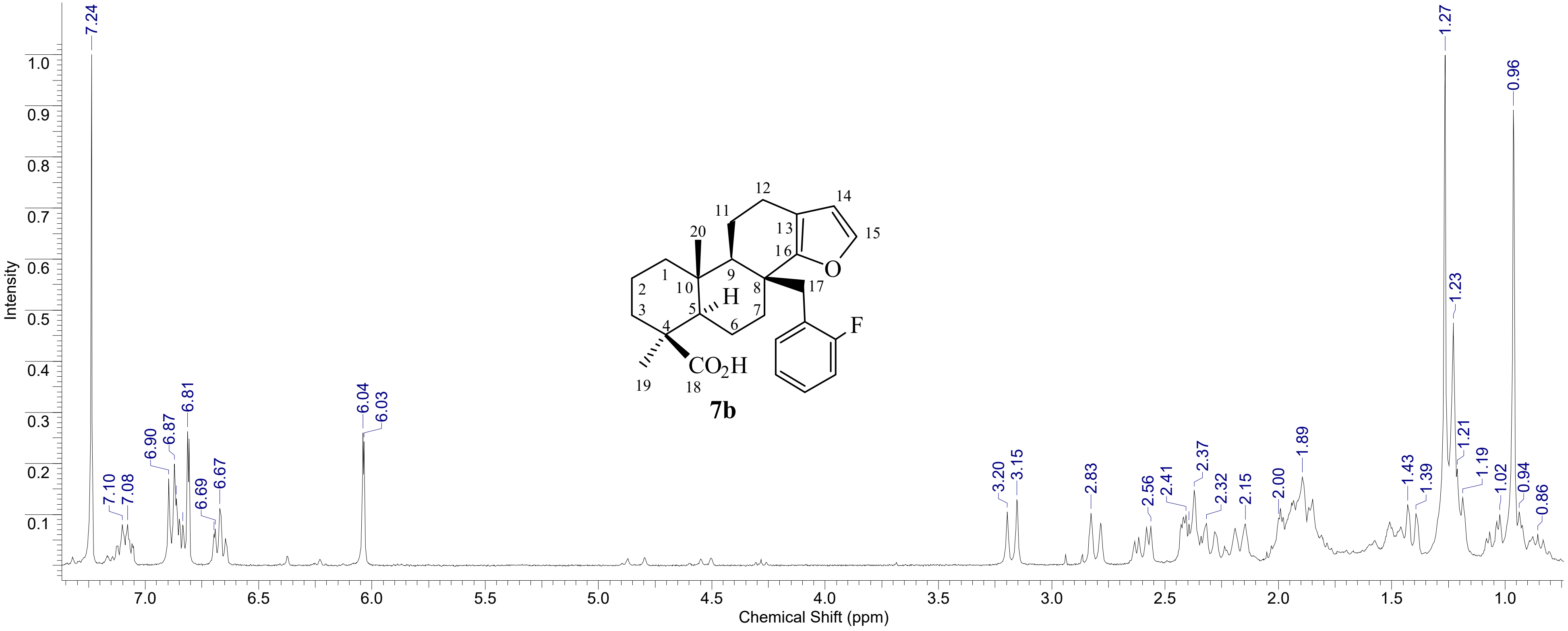

(3bS,5aR,6S,9aR,9bR)-3b-(2-fluorobenzyl)-6,9a-dimethyl-3b,4,5,5a,6,7,8,9,9a,9b,10,11-dodecahydrophenanthro[1,2-b]furan-6-carboxylic acid (**7b**)

(^{13}C NMR, 75 MHz, $CDCl_3$)

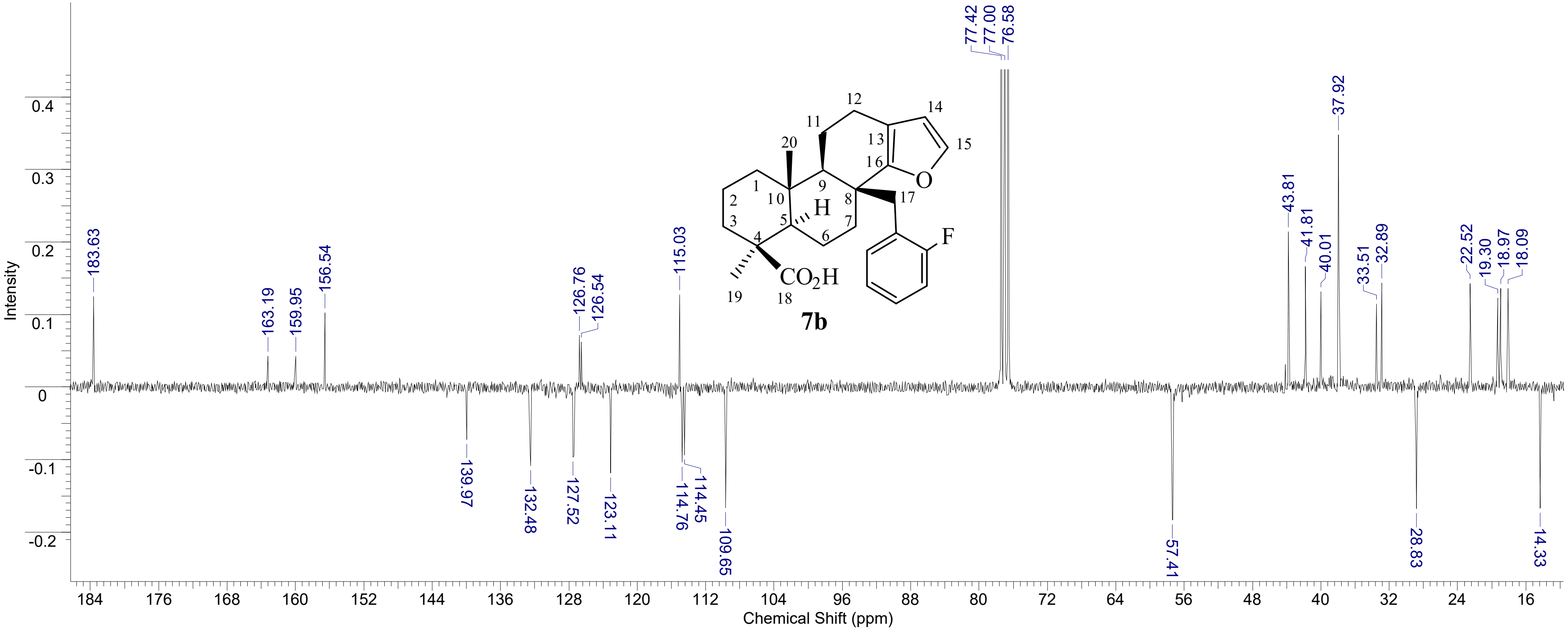

(1S,4aR,5S,8aR)-5-(2-(2-(2-fluorophenyl)furan-3-yl)ethyl)-1,4a-dimethyl-6-methylenedecahydronaphthalene-1-carboxylic acid (8b)

(^{1}H NMR, 400 MHz, $CDCl_3$)

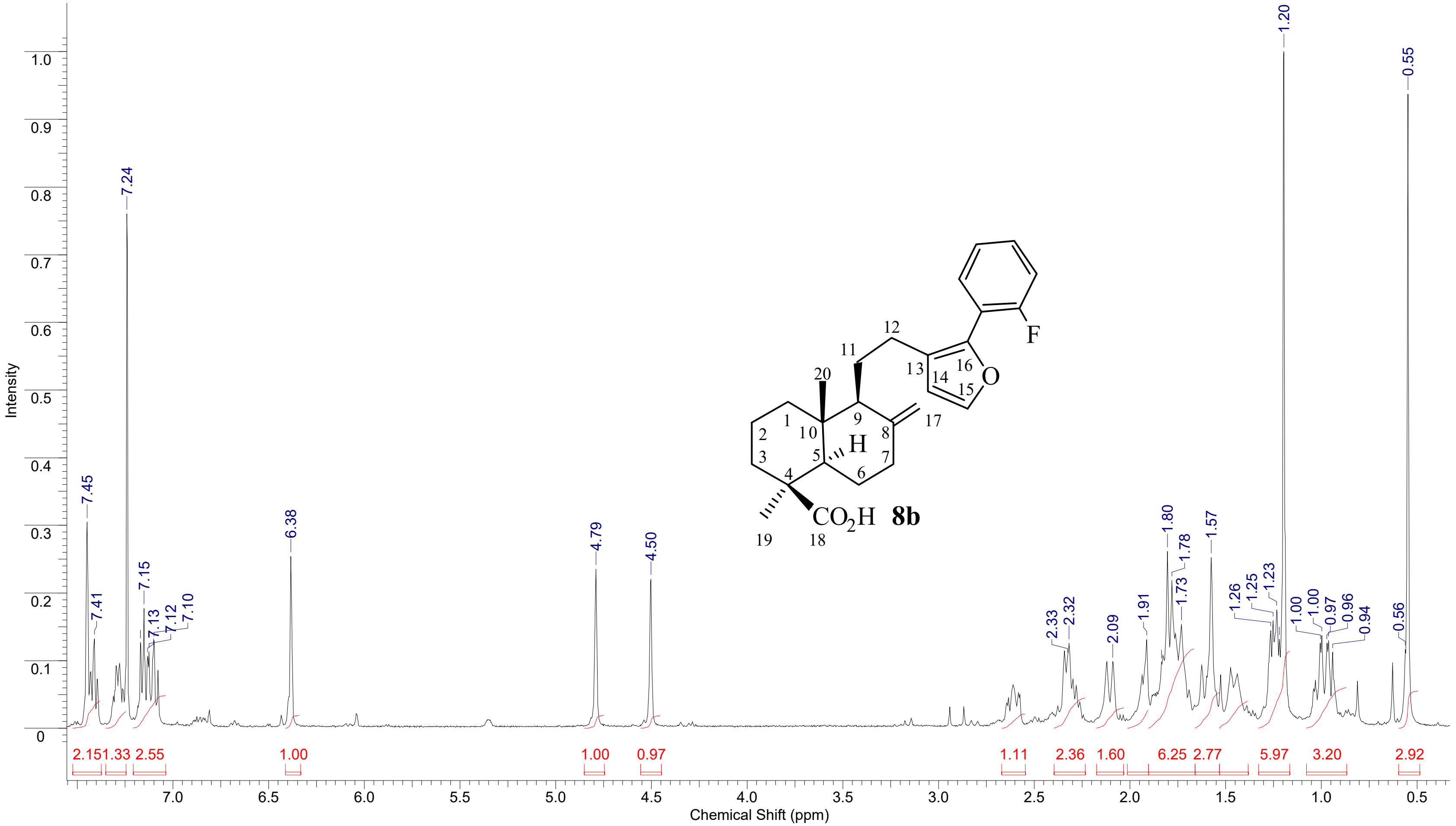

(1S,4aR,5S,8aR)-5-(2-(2-fluorophenyl)furan-3-yl)ethyl)-1,4a-dimethyl-6-methylenedecahydronaphthalene-1-carboxylic acid (8b)

(^{13}C NMR, 126 MHz, $CDCl_3$)

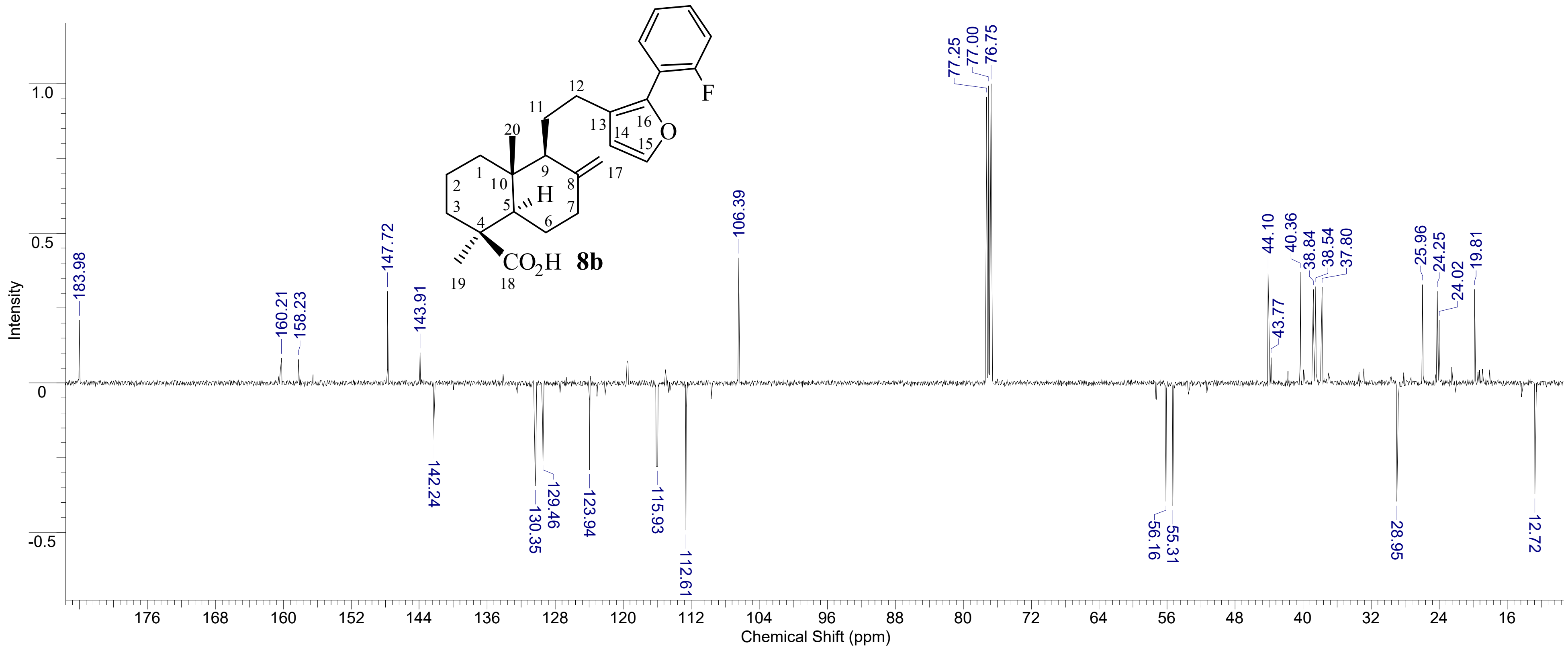

(3bS,5aR,6S,9aR,9bR)-3b-(4-fluorobenzyl)-6,9a-dimethyl-3b,4,5,5a,6,7,8,9,9a,9b,10,11-dodecahydrophenanthro[1,2-b]furan-6-carboxylic acid (7c)

(^{1}H NMR, 400 MHz, $CDCl_3$)

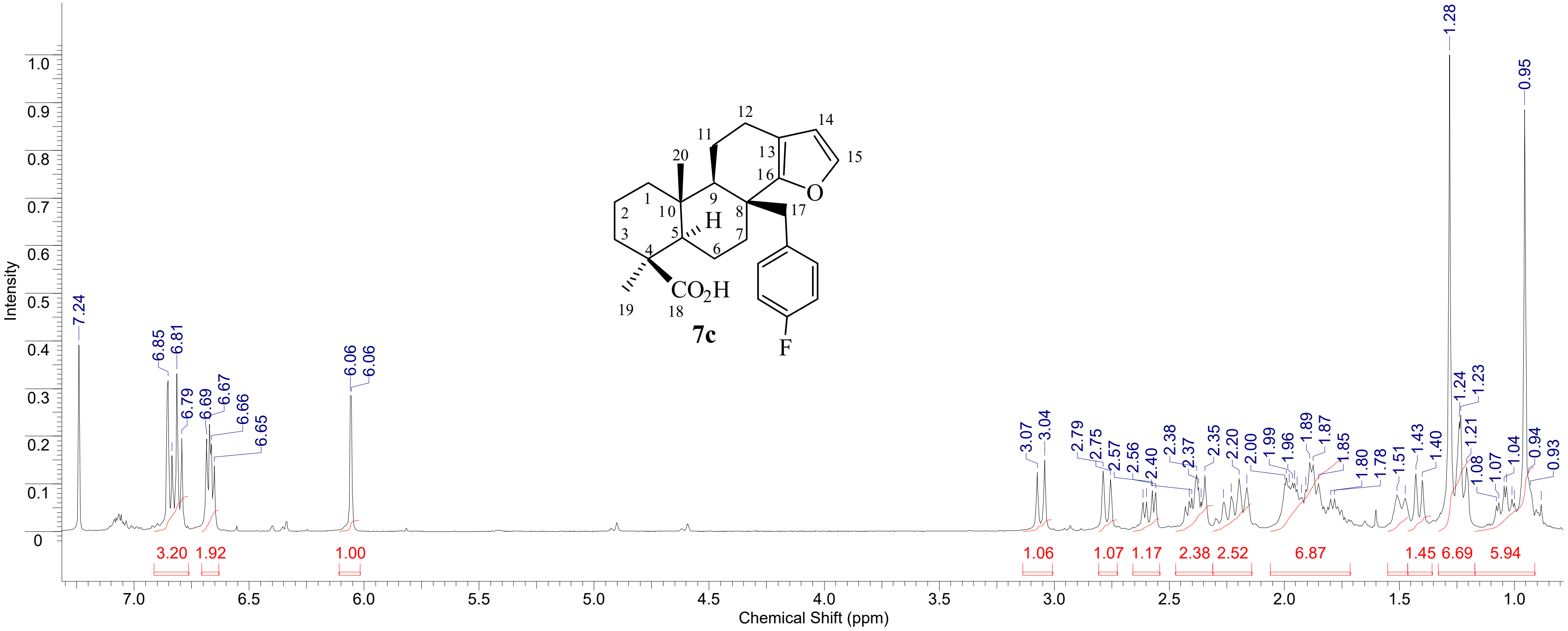

(3bS,5aR,6S,9aR,9bR)-3b-(4-fluorobenzyl)-6,9a-dimethyl-3b,4,5,5a,6,7,8,9,9a,9b,10,11-dodecahydrophenanthro[1,2-b]furan-6-carboxylic acid (7c)

(^{13}C NMR, 126 MHz, $CDCl_3$)

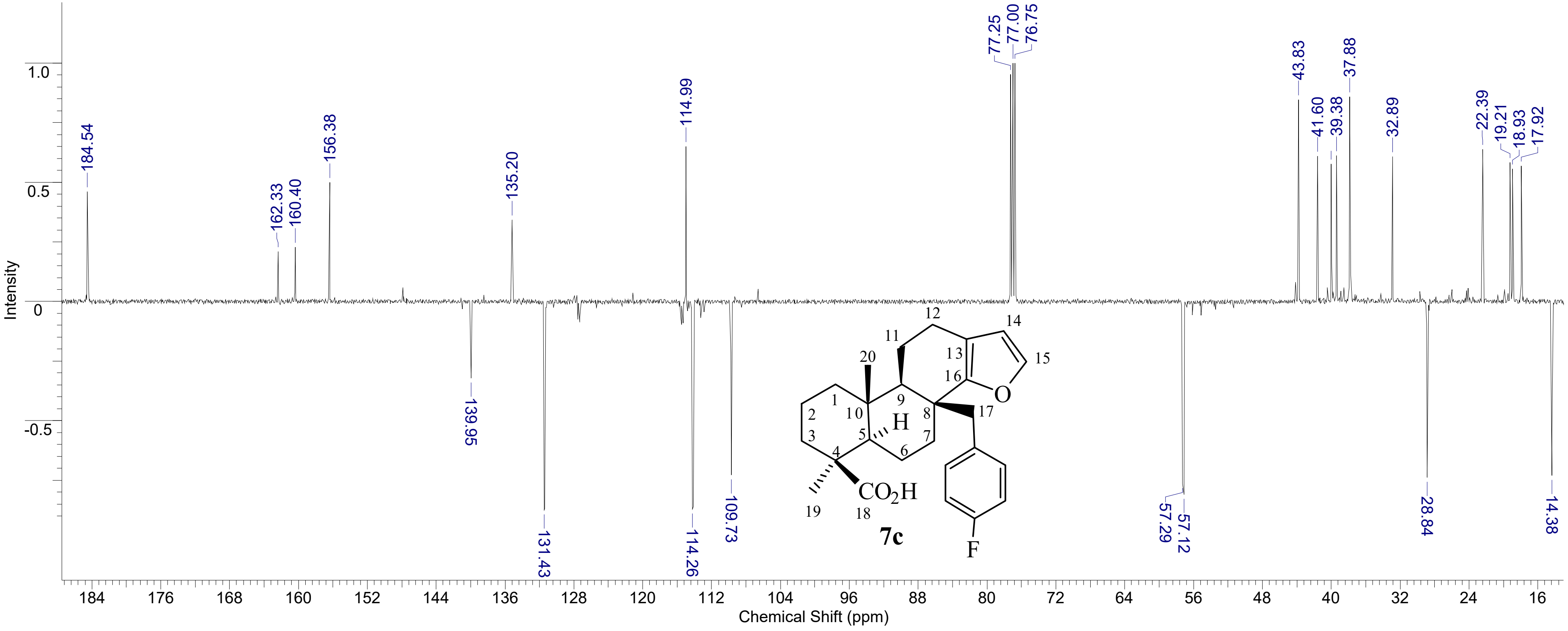

(1S,4aR,5S,8aR)-5-(2-(2-(5-fluorophenyl)furan-3-yl)ethyl)-1,4a-dimethyl-6-methylenedecahydronaphthalene-1-carboxylic acid (8c)

(^{1}H NMR, 400 MHz, $CDCl_3$)

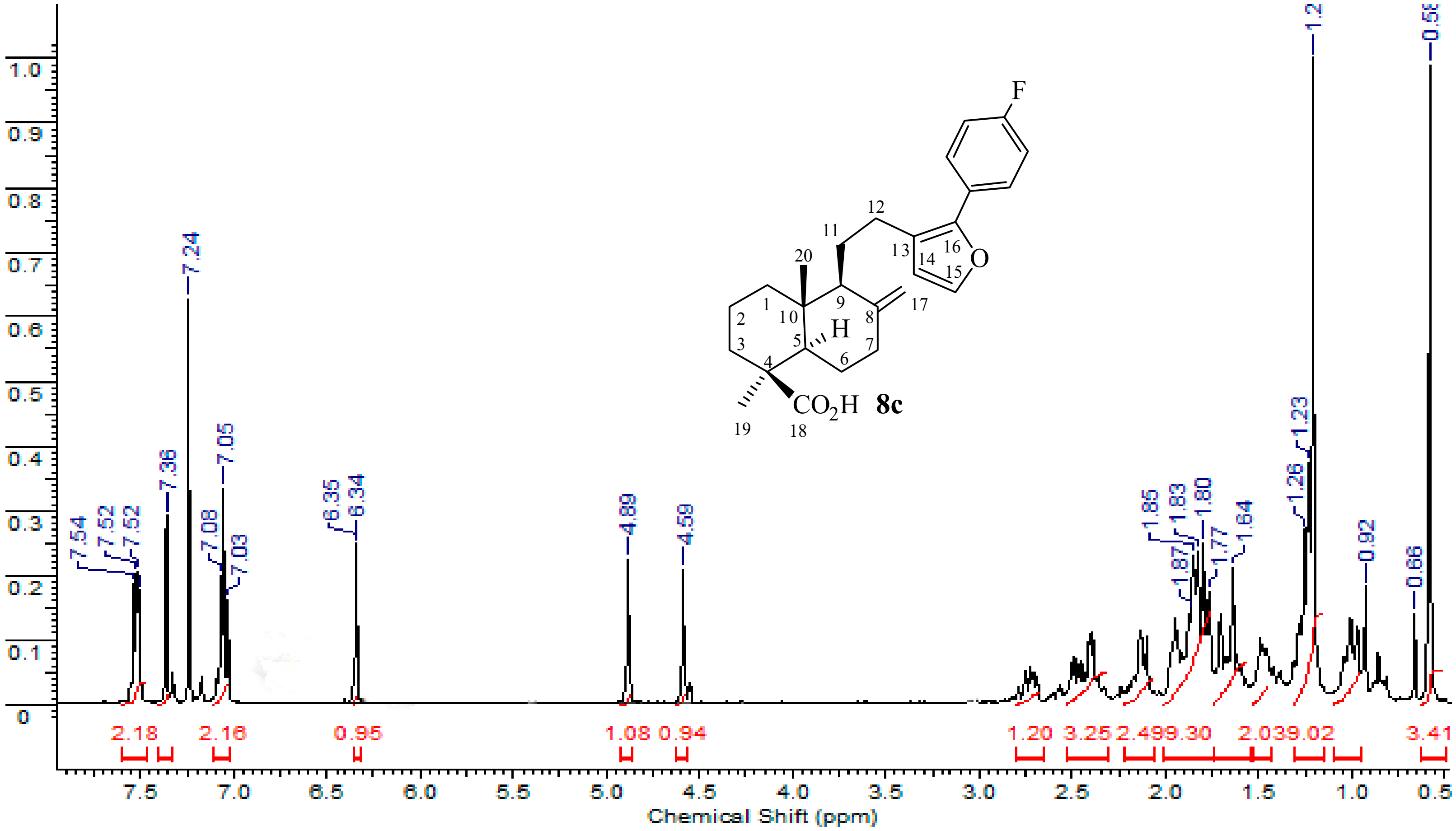

(1S,4aR,5S,8aR)-5-(2-(2-(5-fluorophenyl)furan-3-yl)ethyl)-1,4a-dimethyl-6-methylenedecahydronaphthalene-1-carboxylic acid (8c)

(^{13}C NMR, 75 MHz, $CDCl_3$)

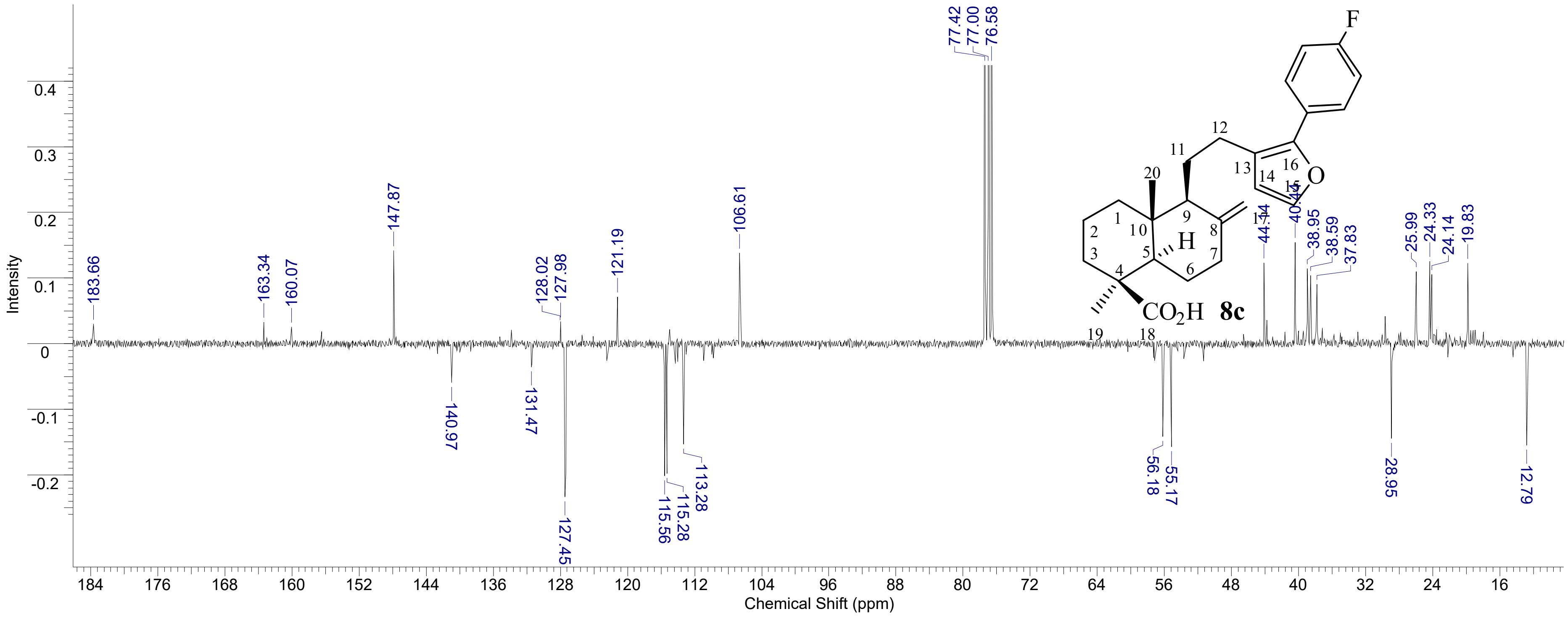

(3bS,5aR,6S,9aR,9bR)-3b-(4-methoxybenzyl)-6,9a-dimethyl-3b,4,5,5a,6,7,8,9,9a,9b,10,11-dodecahydrophenanthro[1,2-b]furan-6-carboxylic acid

(7d) (^{1}H NMR, 400 MHz, $CDCl_3$)

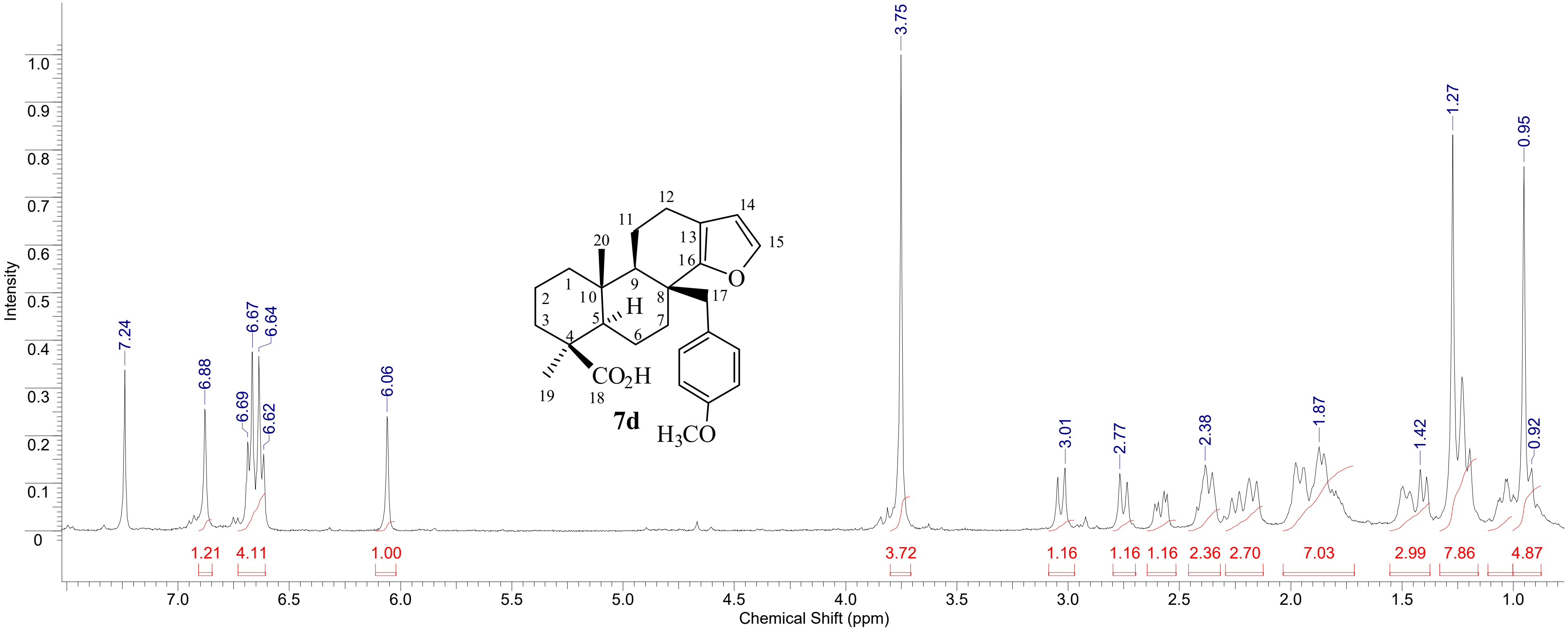

(*3bS,5aR,6S,9aR,9bR)-3b-(4-methoxybenzyl)-6,9a-dimethyl-3b,4,5,5a,6,7,8,9,9a,9b,10,11-dodecahydrophenanthro[1,2-b]furan-6-carboxylic acid*
(7d) (^{13}C NMR, 75 MHz, $CDCl_3$)

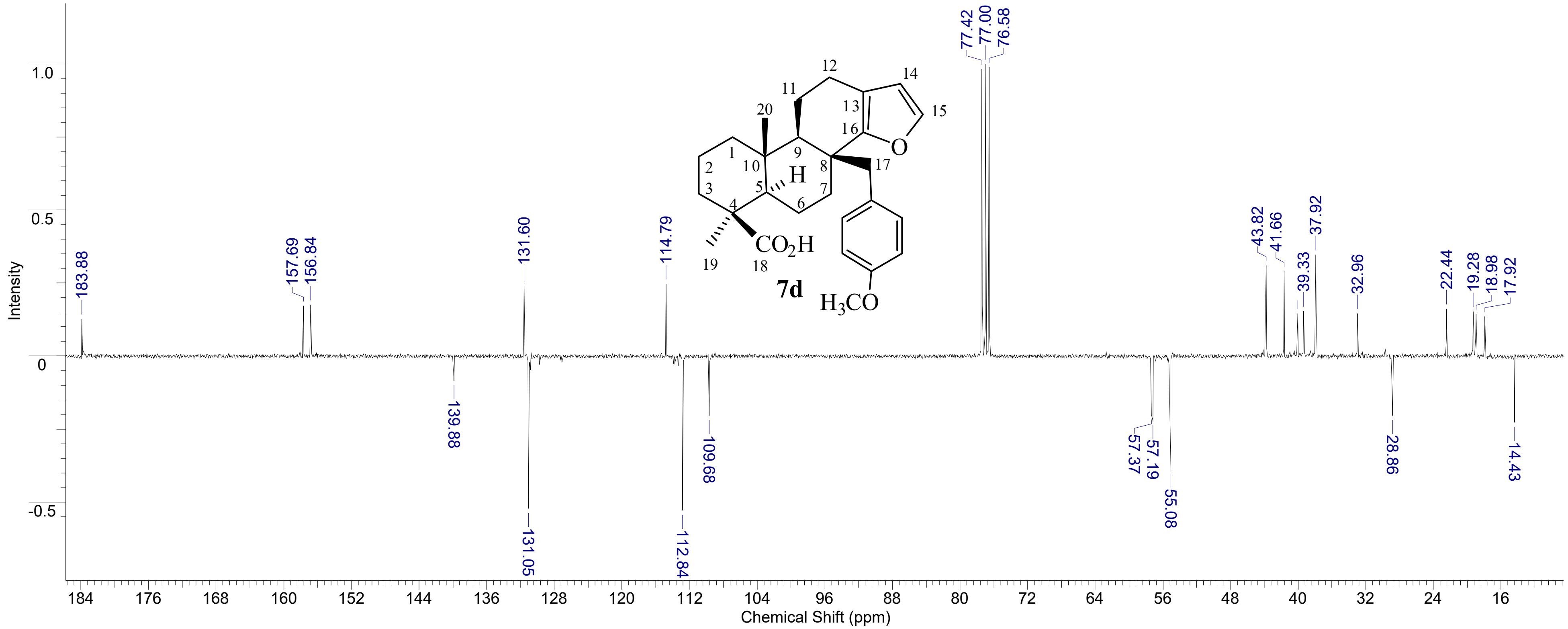

(1S,4aR,5S,8aR)-5-(2-(2-(4-methoxyphenyl)furan-3-yl)ethyl)-1,4a-dimethyl-6-methylenedecahydronaphthalene-1-carboxylic acid (8d)

(^{1}H NMR, 400 MHz, $CDCl_3$)

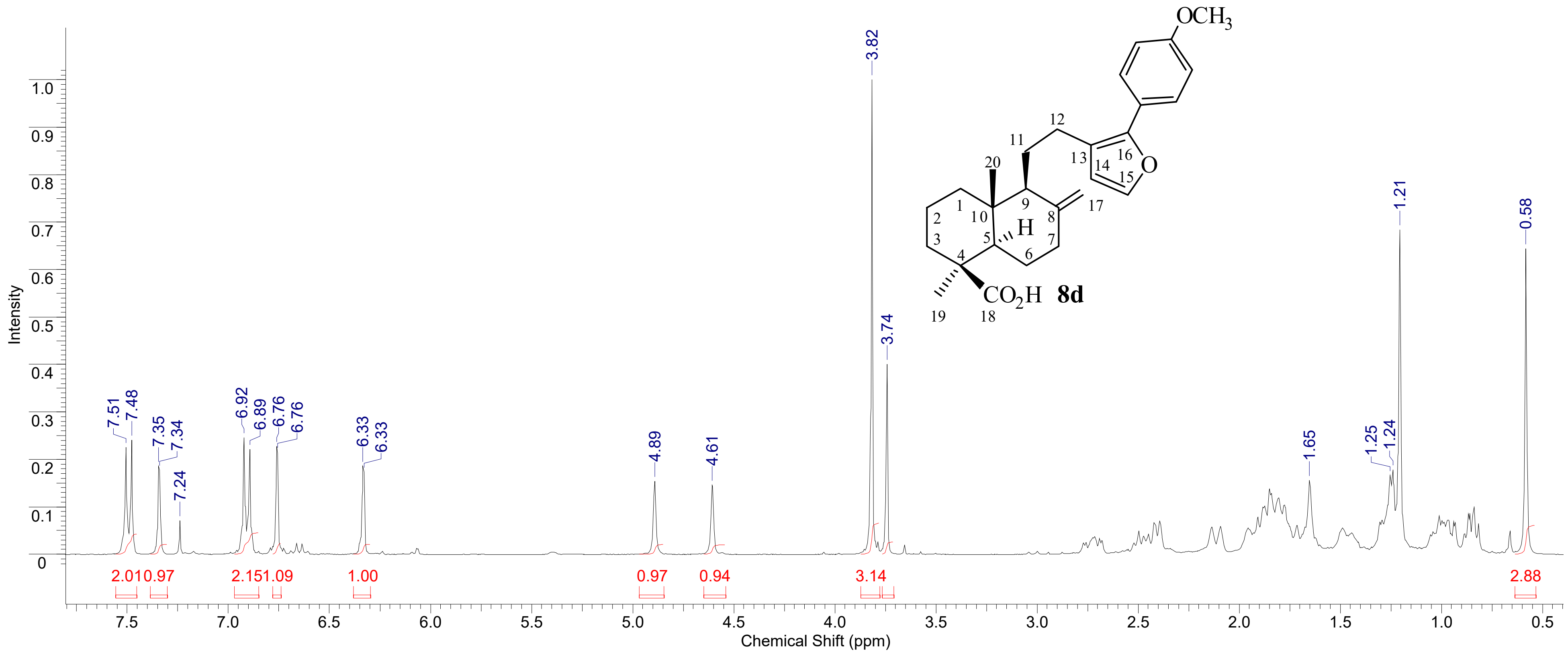

(1S,4aR,5S,8aR)-5-(2-(2-(4-methoxyphenyl)furan-3-yl)ethyl)-1,4a-dimethyl-6-methylenedecahydronaphthalene-1-carboxylic acid (8d)

(^{13}C NMR, 75 MHz, $CDCl_3$)

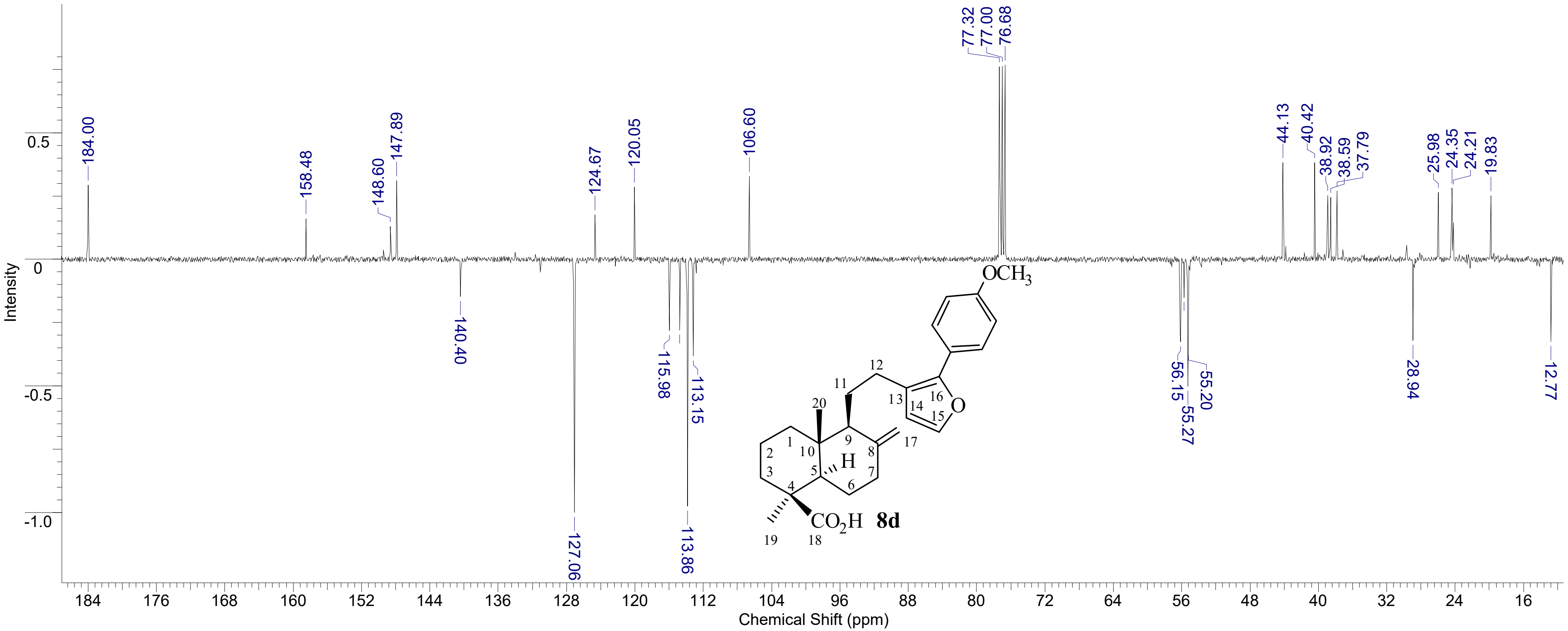

(5aR,6S,9aR,9bR)-6,9a-dimethyl-3b-(2-methylbenzyl)-3b,4,5,5a,6,7,8,9,9a,9b,10,11-dodecahydrophenanthro[1,2-b]furan-6-carboxylic acid (7i)

(^{1}H NMR, 400 MHz, $CDCl_3$)

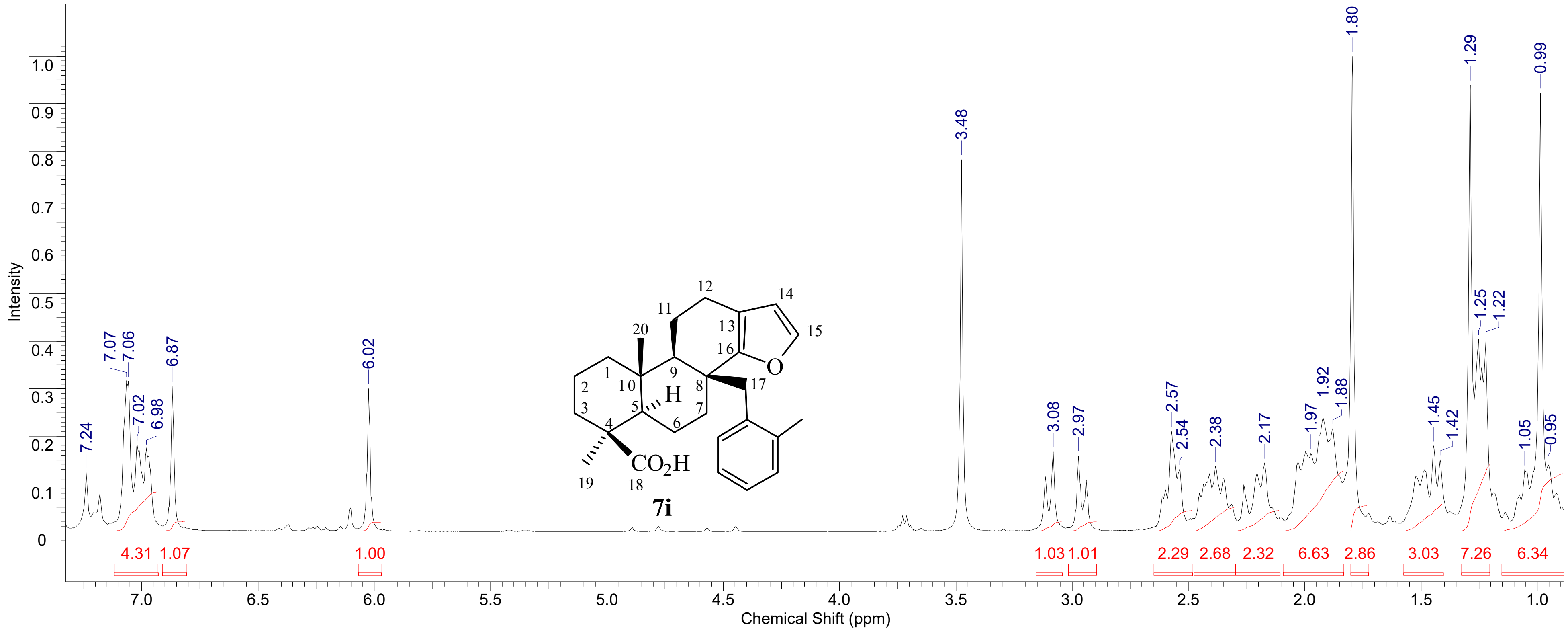

(5aR,6S,9aR,9bR)-6,9a-dimethyl-3b-(2-methylbenzyl)-3b,4,5,5a,6,7,8,9,9a,9b,10,11-dodecahydrophenanthro[1,2-b]furan-6-carboxylic acid (7i)

(^{13}C NMR, 126 MHz, $CDCl_3$)

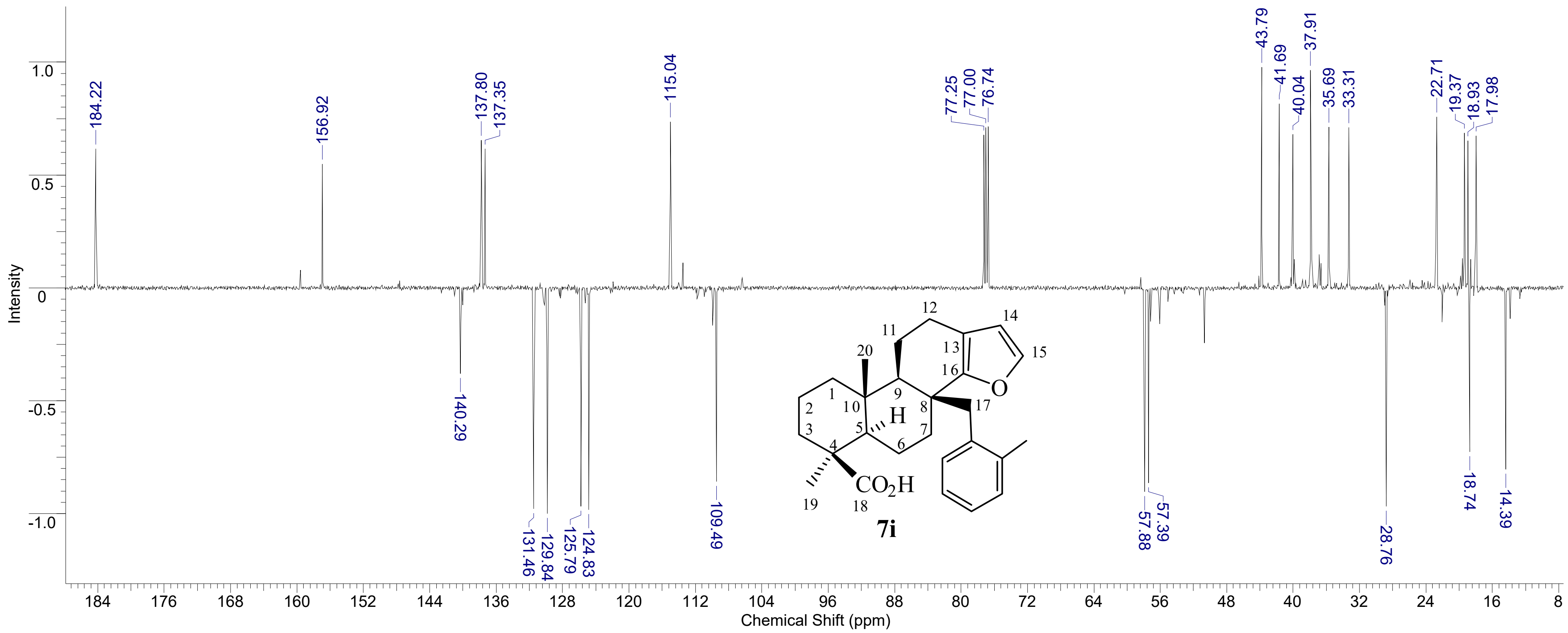

(*5aR,6S,9aR,9bR)-6,9a-dimethyl-3b-(3,4,5-trimethoxybenzyl)-3b,4,5,5a,6,7,8,9,9a,9b,10,11-dodecahydrophenanthro[1,2-b]furan-6-carboxylic acid*

(7j) (^{1}H NMR, 300 MHz, $CDCl_3$)

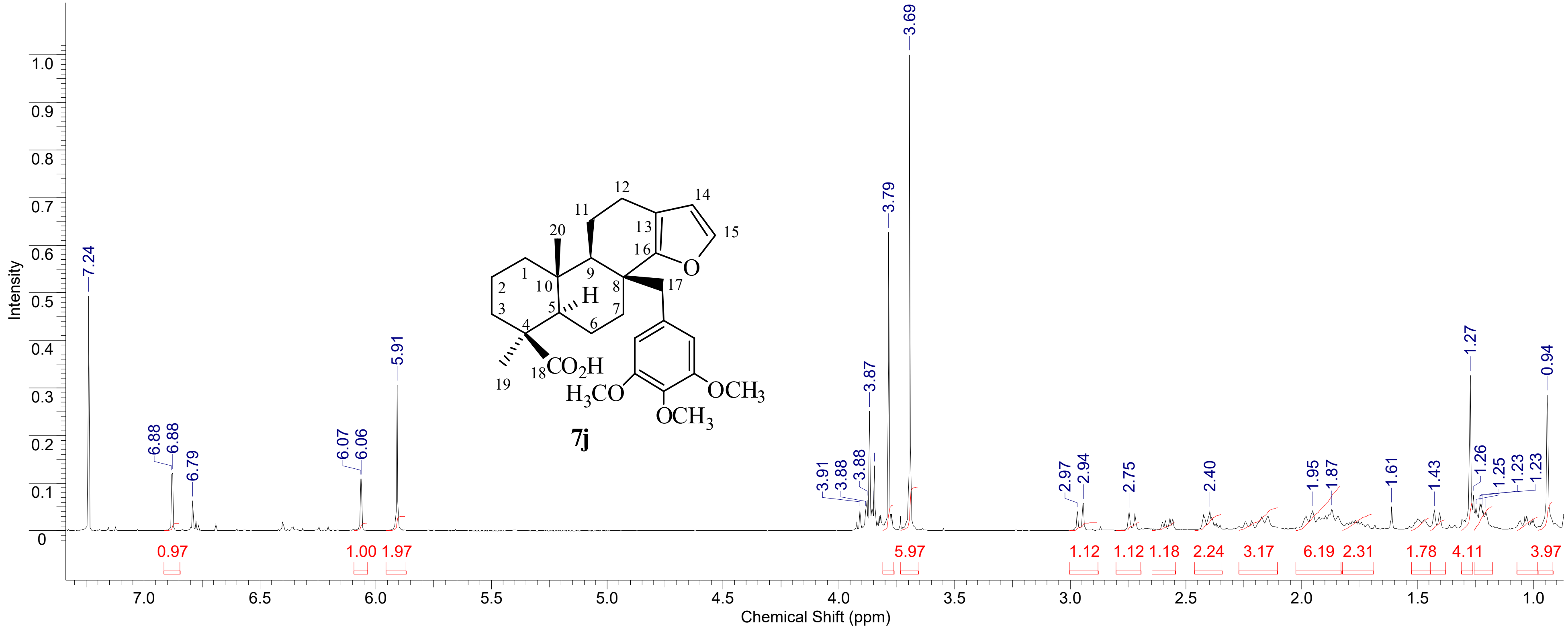

(5aR,6S,9aR,9bR)-6,9a-dimethyl-3b-(3,4,5-trimethoxybenzyl)-3b,4,5,5a,6,7,8,9,9a,9b,10,11-dodecahydrophenanthro[1,2-b]furan-6-carboxylic acid
(7j) (^{13}C NMR, 126 MHz, $CDCl_3$)

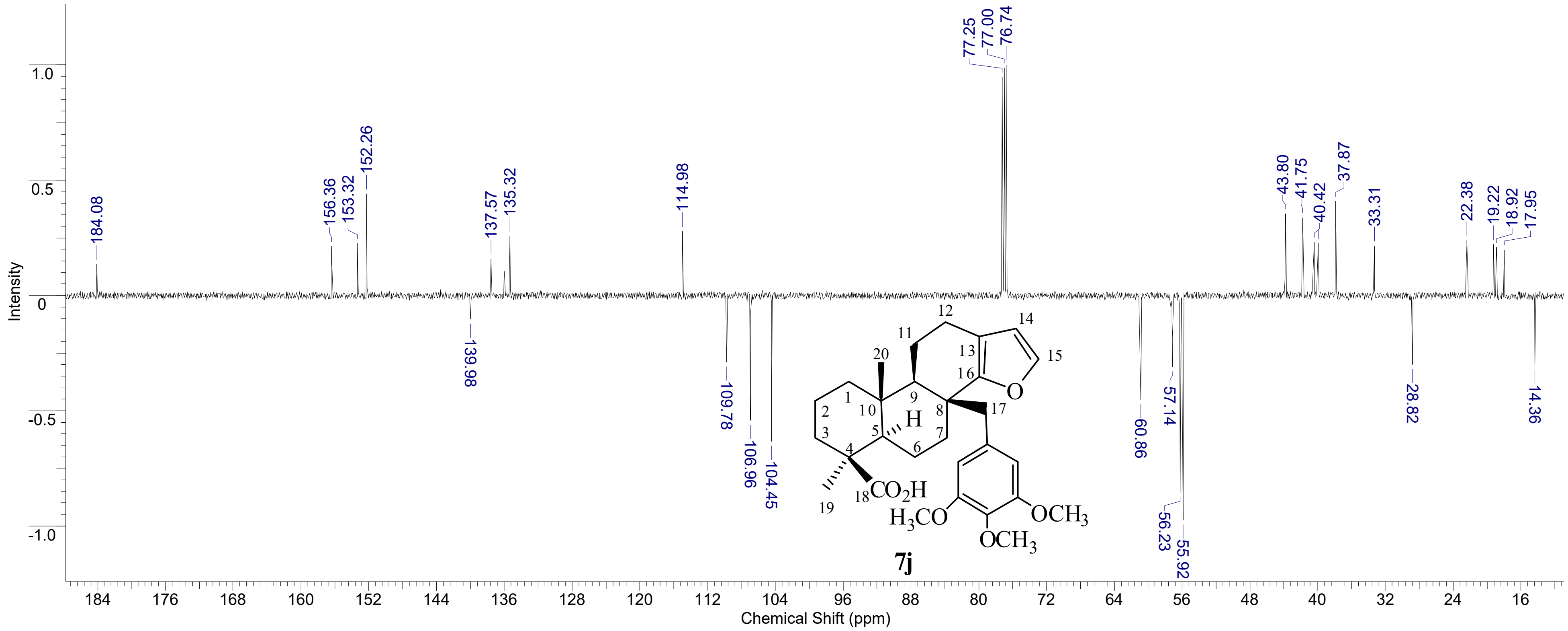

(1S,4aR,5S,8aR)-1,4a-dimethyl-6-methylene-5-(2-(2-(3,4,5-trimethoxyphenyl)furan-3-yl)ethyl)decahydronaphthalene-1-carboxylic acid (8j)

(^{1}H NMR, 300 MHz, $CDCl_3$)

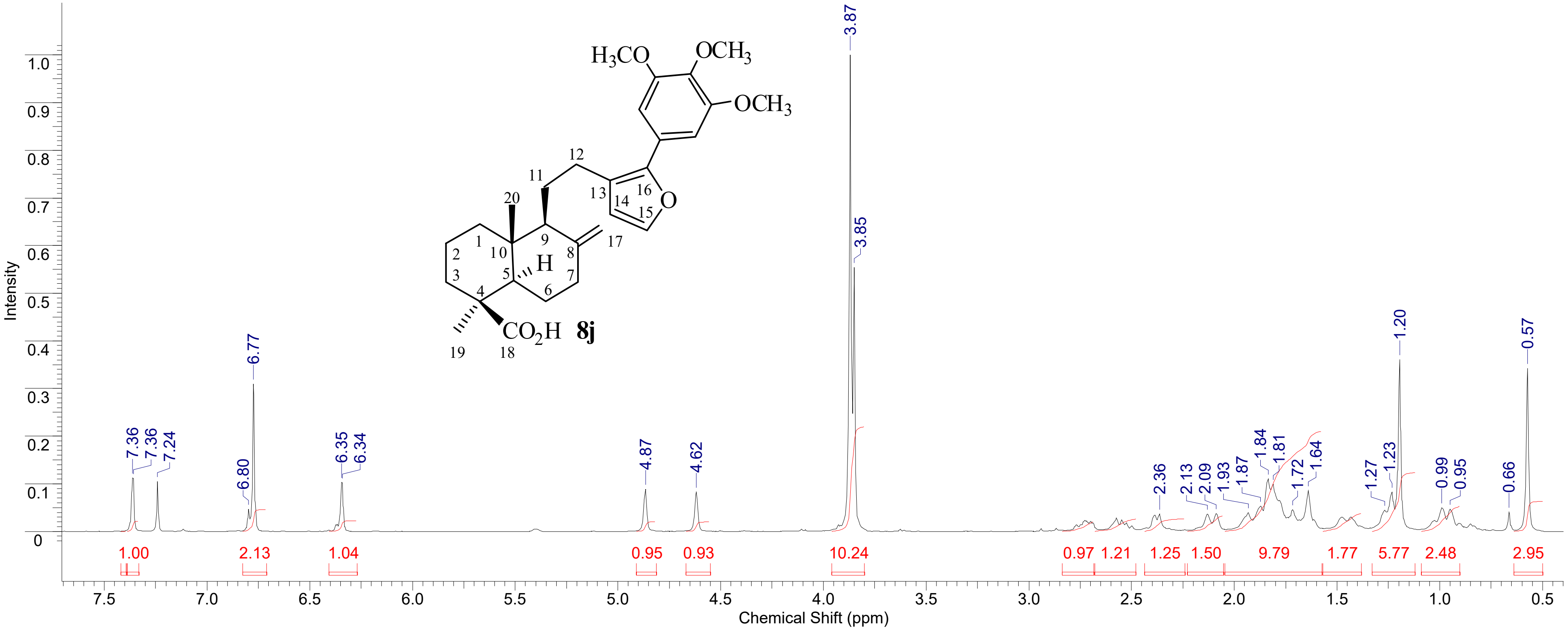

(1S,4aR,5S,8aR)-1,4a-dimethyl-6-methylene-5-(2-(2-(3,4,5-trimethoxyphenyl)furan-3-yl)ethyl)decahydronaphthalene-1-carboxylic acid (8j)

(^{13}C NMR, 151 MHz, $CDCl_3$)

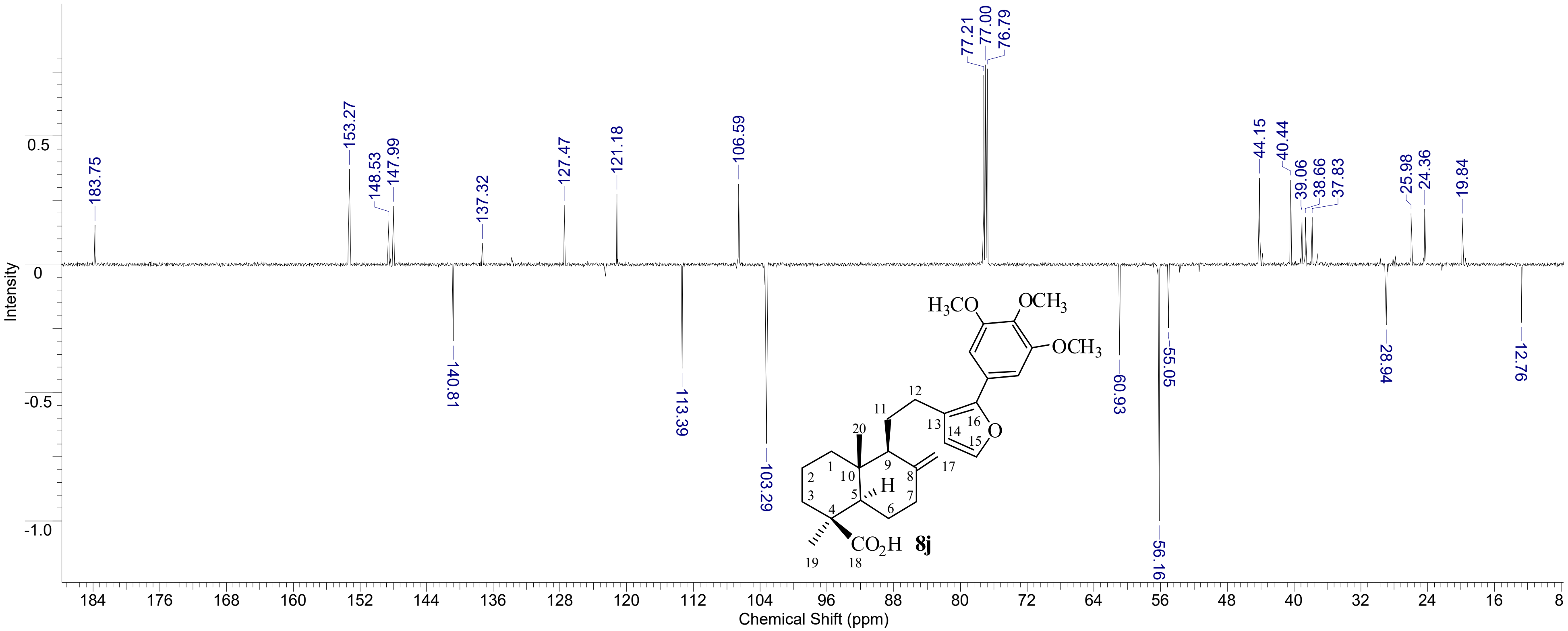

(5aR,6S,9aR,9bR)-6,9a-dimethyl-3b-(4-(trifluoromethyl)benzyl)-3b,4,5,5a,6,7,8,9,9a,9b,10,11-dodecahydrophenanthro[1,2-b]furan-6-carboxylic acid (**7e**) (^{1}H NMR, 400 MHz, $CDCl_3$)

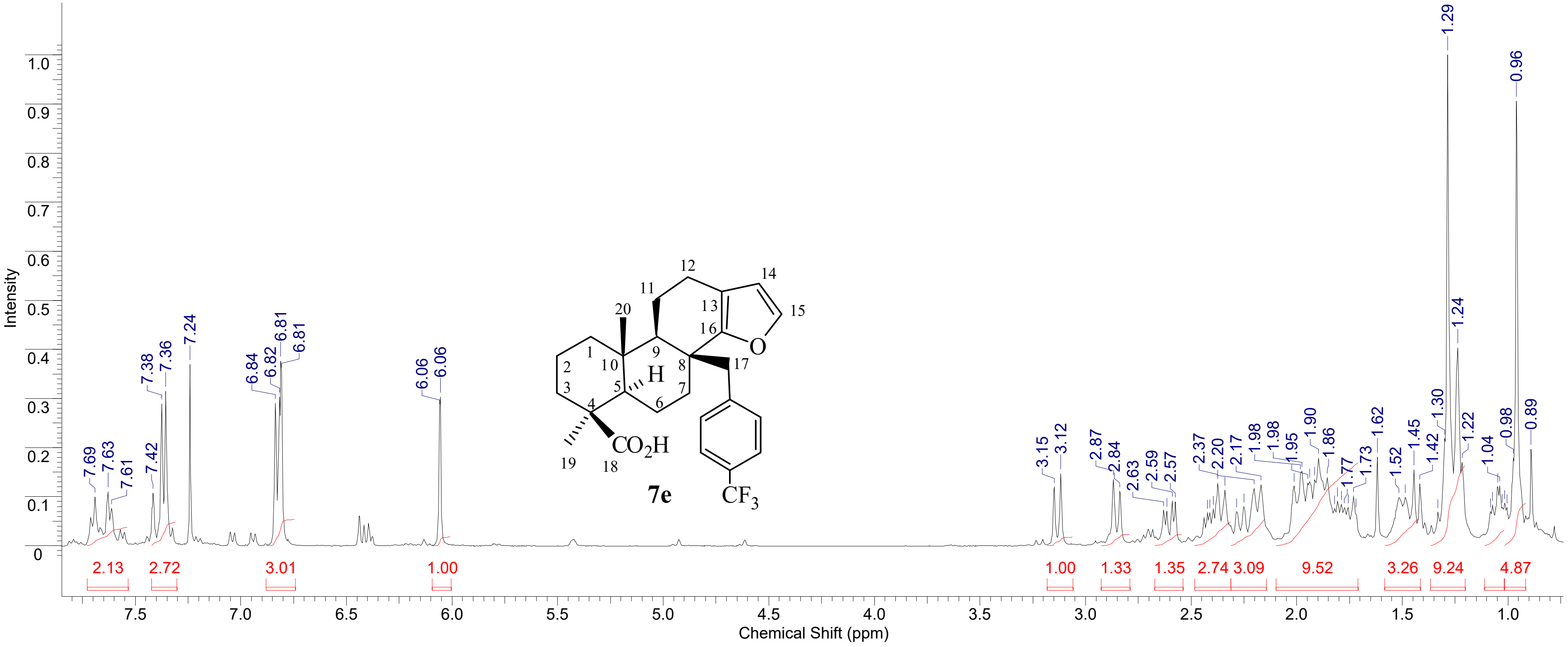

(5aR,6S,9aR,9bR)-6,9a-dimethyl-3b-(4-(trifluoromethyl)benzyl)-3b,4,5,5a,6,7,8,9,9a,9b,10,11-dodecahydrophenanthro[1,2-b]furan-6-carboxylic acid (**7e**) (^{13}C NMR, 126 MHz, $CDCl_3$)

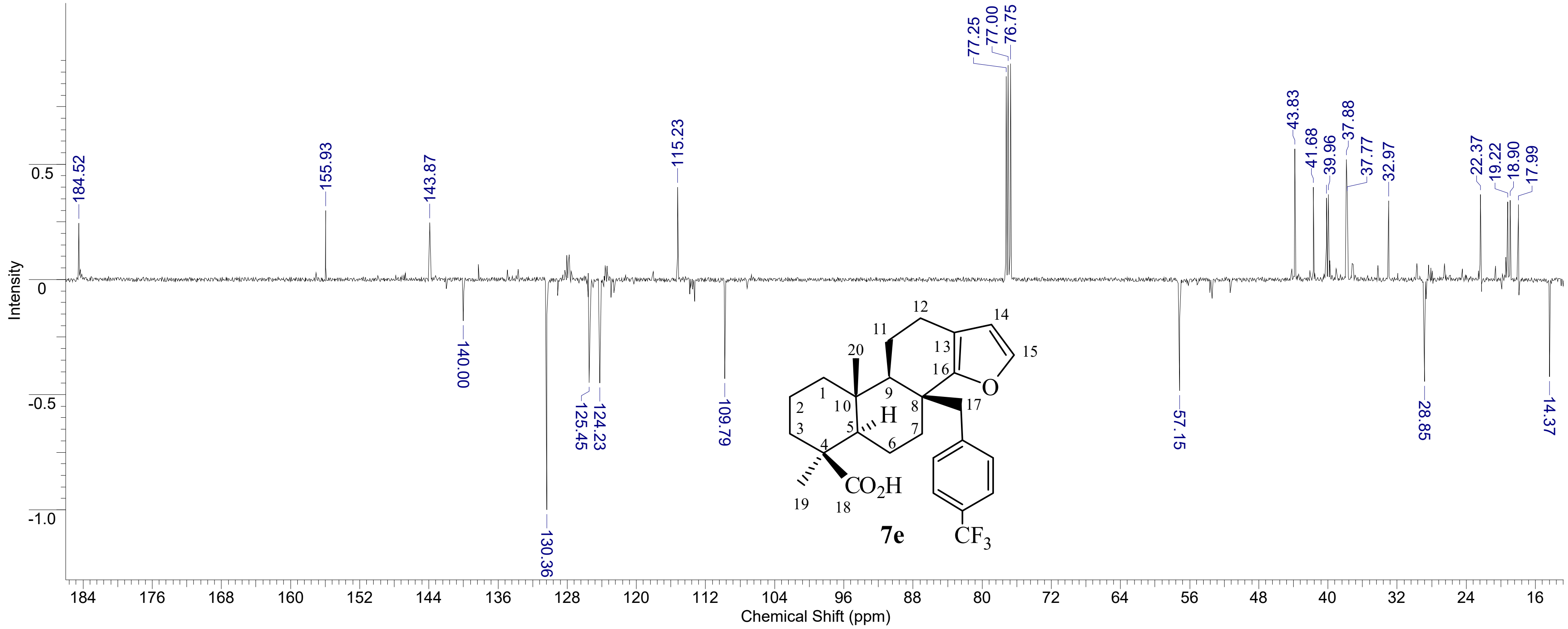

(3bS,5aR,6S,9aR,9bR)-3b-(4-cyanobenzyl)-6,9a-dimethyl-3b,4,5,5a,6,7,8,9,9a,9b,10,11-dodecahydrophenanthro[1,2-b]furan-6-carboxylic acid (7f)

(^{1}H NMR, 600 MHz, $CDCl_3$)

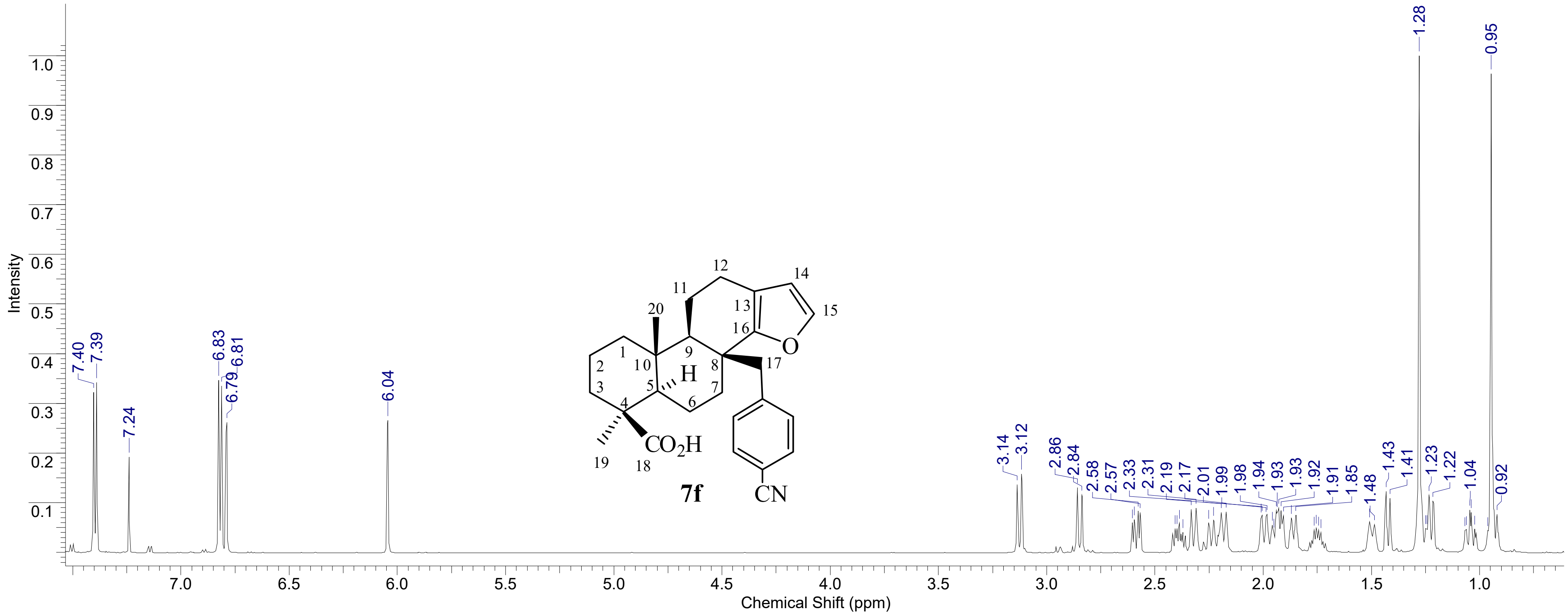

(3bS,5aR,6S,9aR,9bR)-3b-(4-cyanobenzyl)-6,9a-dimethyl-3b,4,5,5a,6,7,8,9,9a,9b,10,11-dodecahydrophenanthro[1,2-b]furan-6-carboxylic acid (7f)

(^{13}C NMR, 151 MHz, $CDCl_3$)

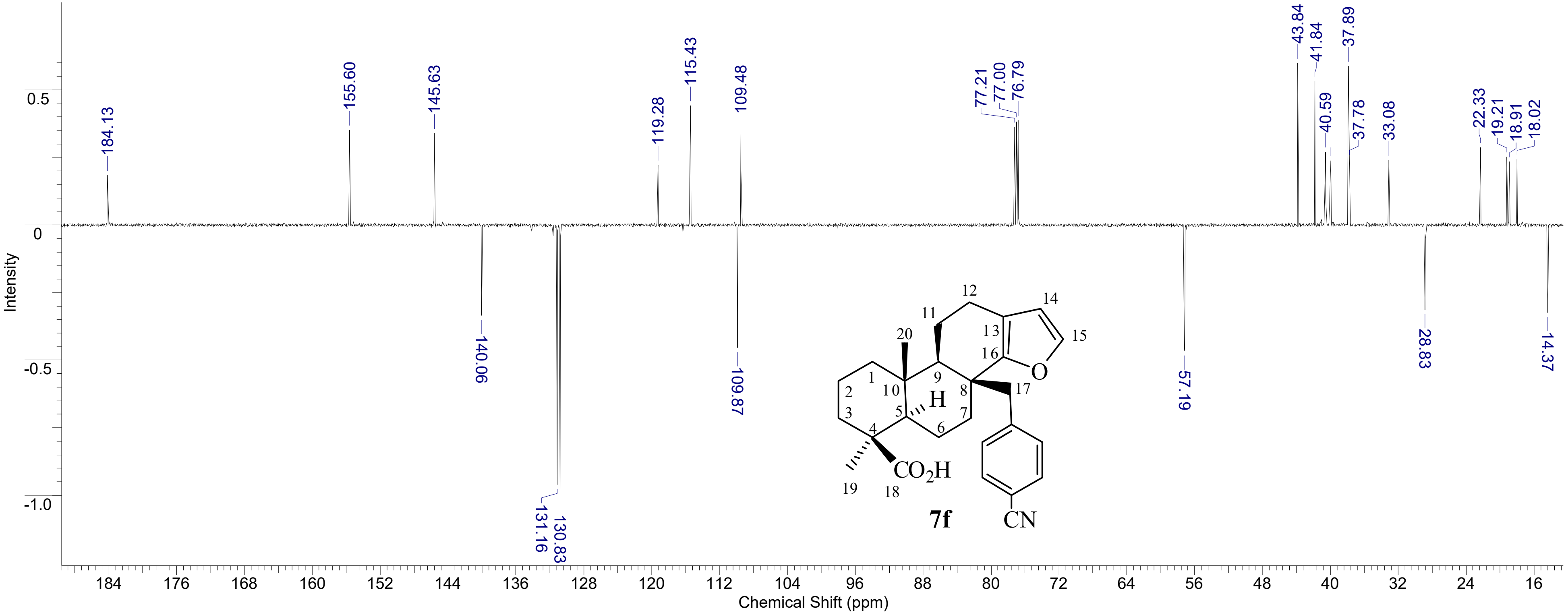

(3bS,5aR,6S,9aR,9bR)-3b-(3-fluorobenzyl)-6,9a-dimethyl-3b,4,5,5a,6,7,8,9,9a,9b,10,11-dodecahydrophenanthro[1,2-b]furan-6-carboxylic acid (**7g**)

(^{1}H NMR, 300 MHz, $CDCl_3$)

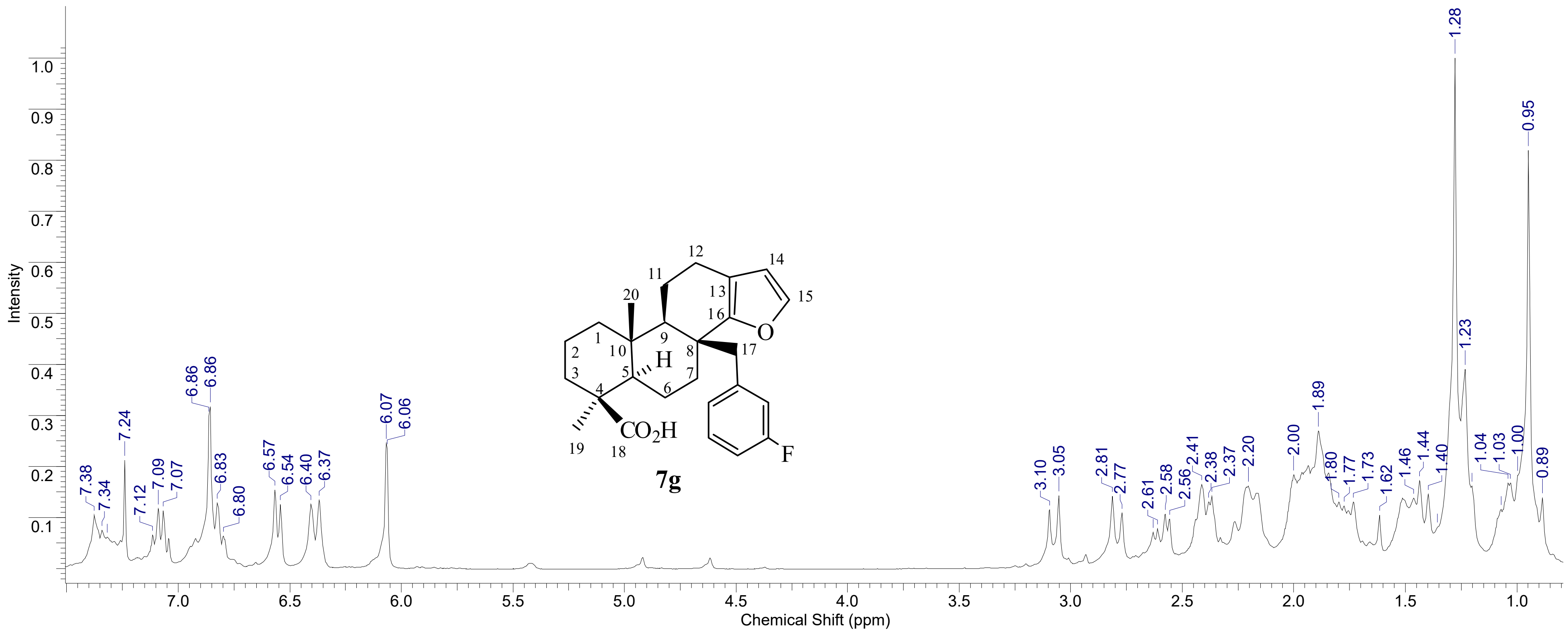

***(3bS,5aR,6S,9aR,9bR)-3b-(3-fluorobenzyl)-6,9a-dimethyl-3b,4,5,5a,6,7,8,9,9a,9b,10,11-dodecahydrophenanthro[1,2-b]furan-6-carboxylic acid* (7g)**

(^{13}C NMR, 126 MHz, $CDCl_3$)

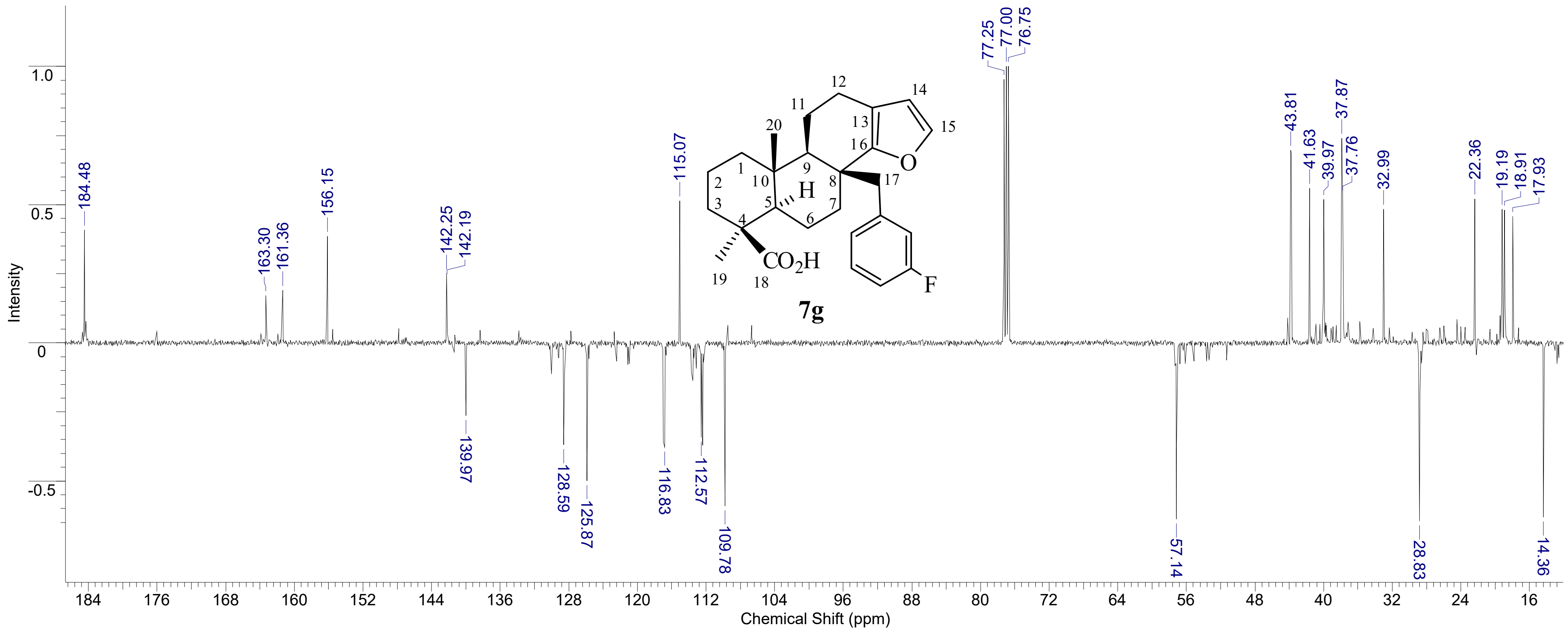

(3bS,5aR,6S,9aR,9bR)-3b-(3-methoxybenzyl)-6,9a-dimethyl-3b,4,5,5a,6,7,8,9,9a,9b,10,11-dodecahydrophenanthro[1,2-b]furan-6-carboxylic acid

(**7h**) (^{1}H NMR, 500 MHz, $CDCl_3$)

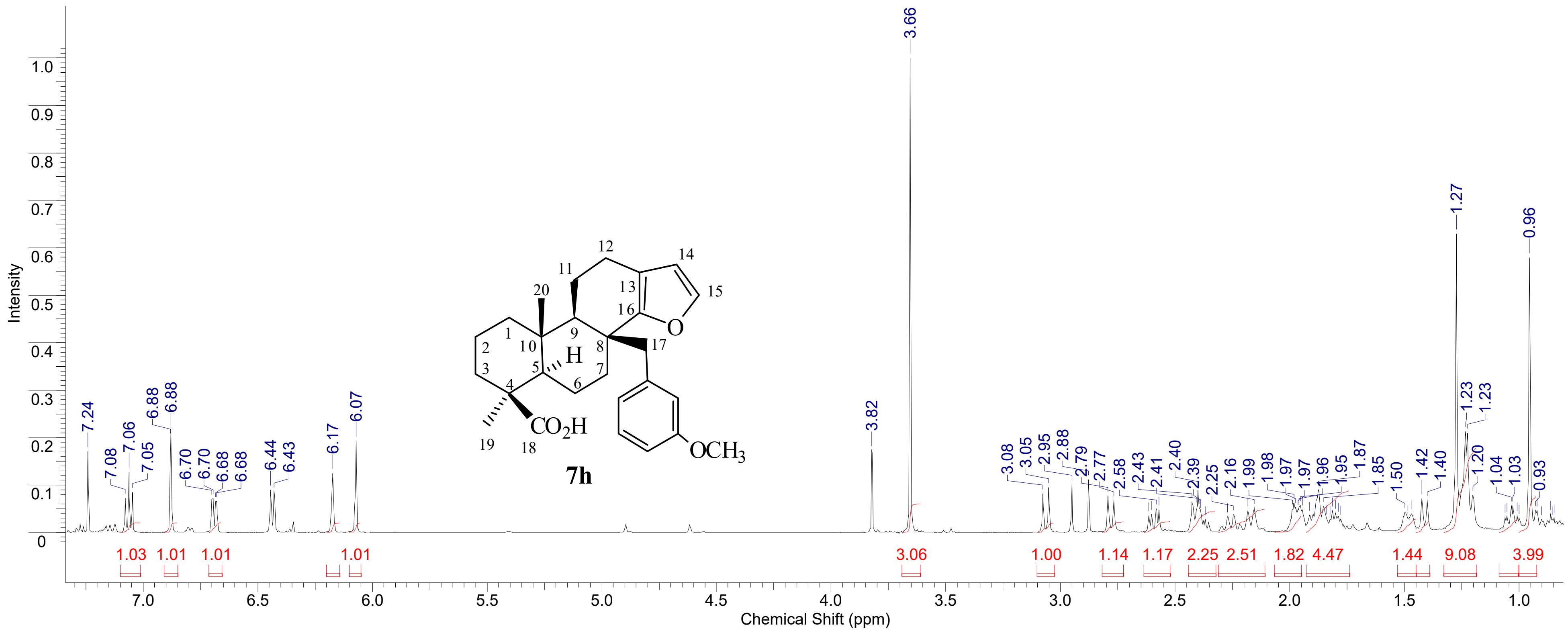

(3bS,5aR,6S,9aR,9bR)-3b-(3-methoxybenzyl)-6,9a-dimethyl-3b,4,5,5a,6,7,8,9,9a,9b,10,11-dodecahydrophenanthro[1,2-b]furan-6-carboxylic acid

(**7h**) (^{13}C NMR, 126 MHz, $CDCl_3$)

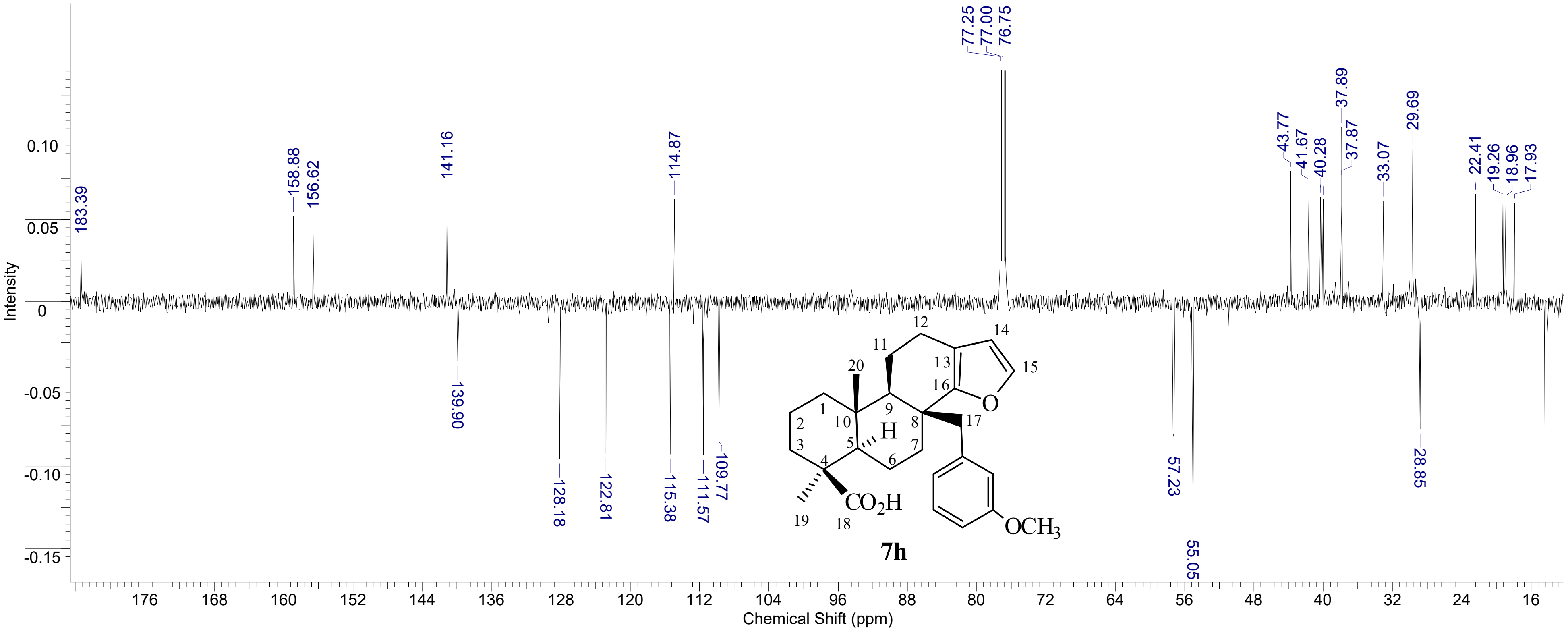

(1S,4aR,5S,8aR)-5-(2-(2-bromo-5-formylfuran-3-yl)ethyl)-1,4a-dimethyl-6-methylenedecahydronaphthalene-1-carboxylic acid (9)

(^{1}H NMR, 300 MHz, $CDCl_3$)

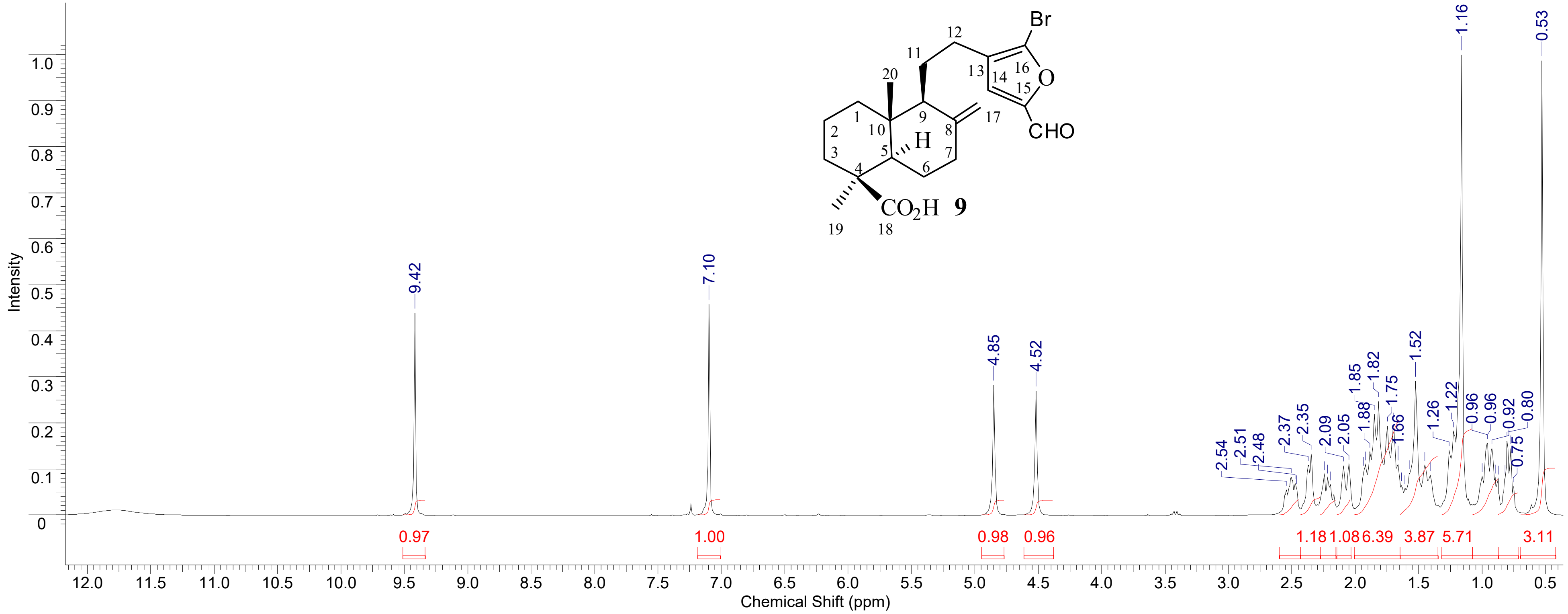

(1S,4aR,5S,8aR)-5-(2-(2-bromo-5-formylfuran-3-yl)ethyl)-1,4a-dimethyl-6-methylenedecahydronaphthalene-1-carboxylic acid (9)

(^{13}C NMR, 126 MHz, $CDCl_3$)

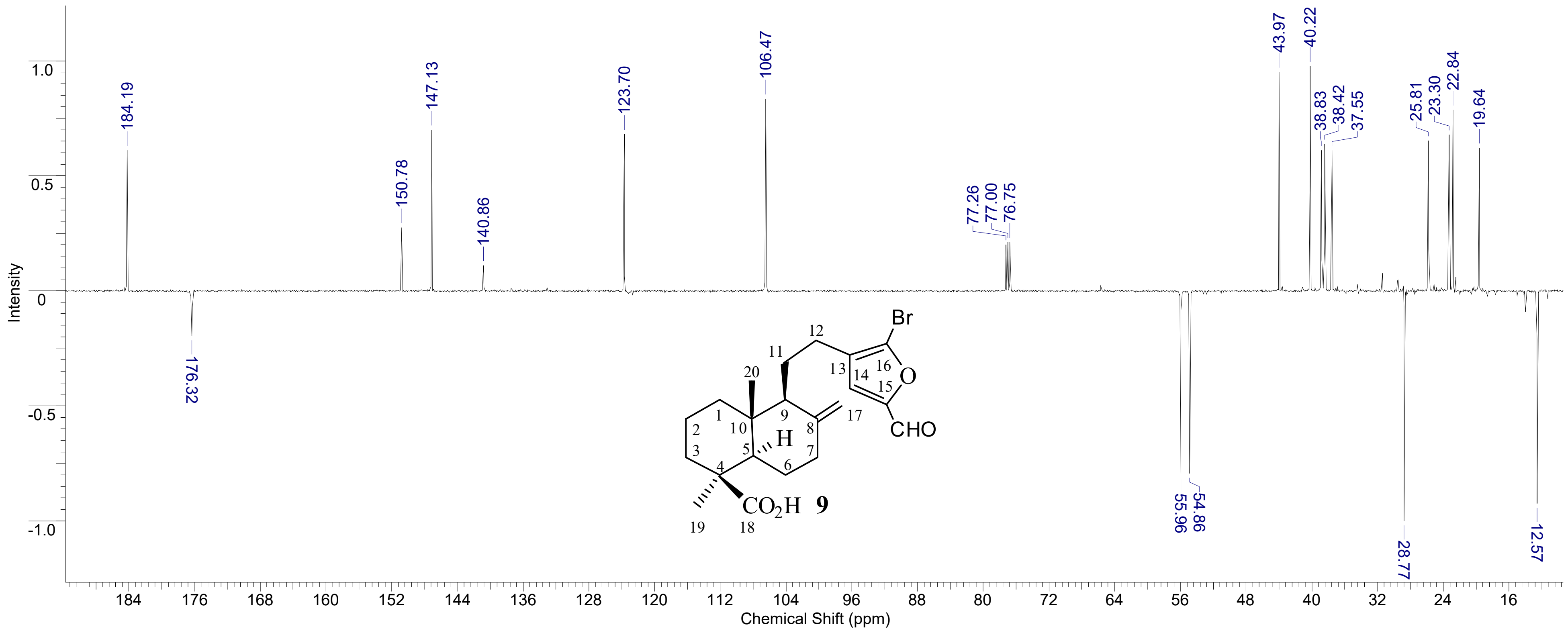

(1S,4aR,5S,8aR)-5-(2-(2-bromo-5-cyanofuran-3-yl)ethyl)-1,4a-dimethyl-6-methylenedecahydronaphthalene-1-carboxylic acid (10)

(^{1}H NMR, 300 MHz, $CDCl_3$)

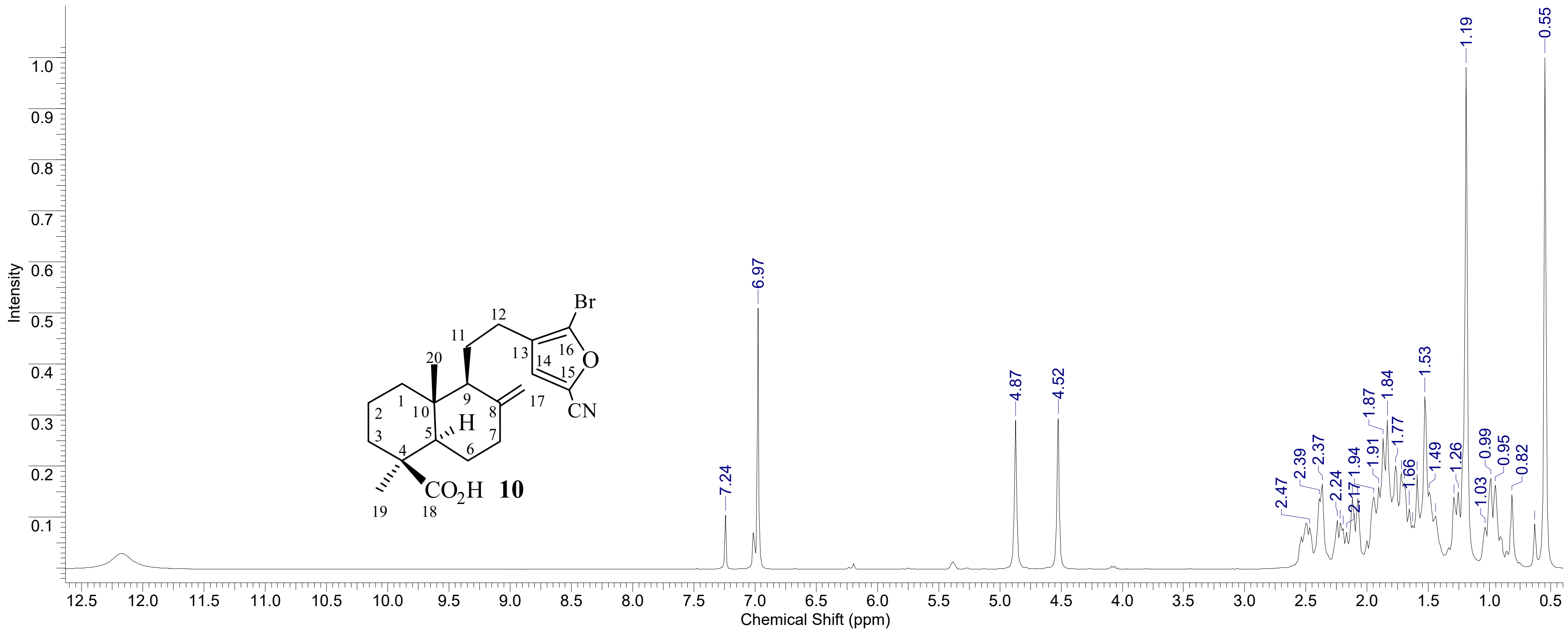

(1S,4aR,5S,8aR)-5-(2-(2-bromo-5-cyanofuran-3-yl)ethyl)-1,4a-dimethyl-6-methylenedecahydronaphthalene-1-carboxylic acid (10)

(^{13}C NMR, 75 MHz, $CDCl_3$)

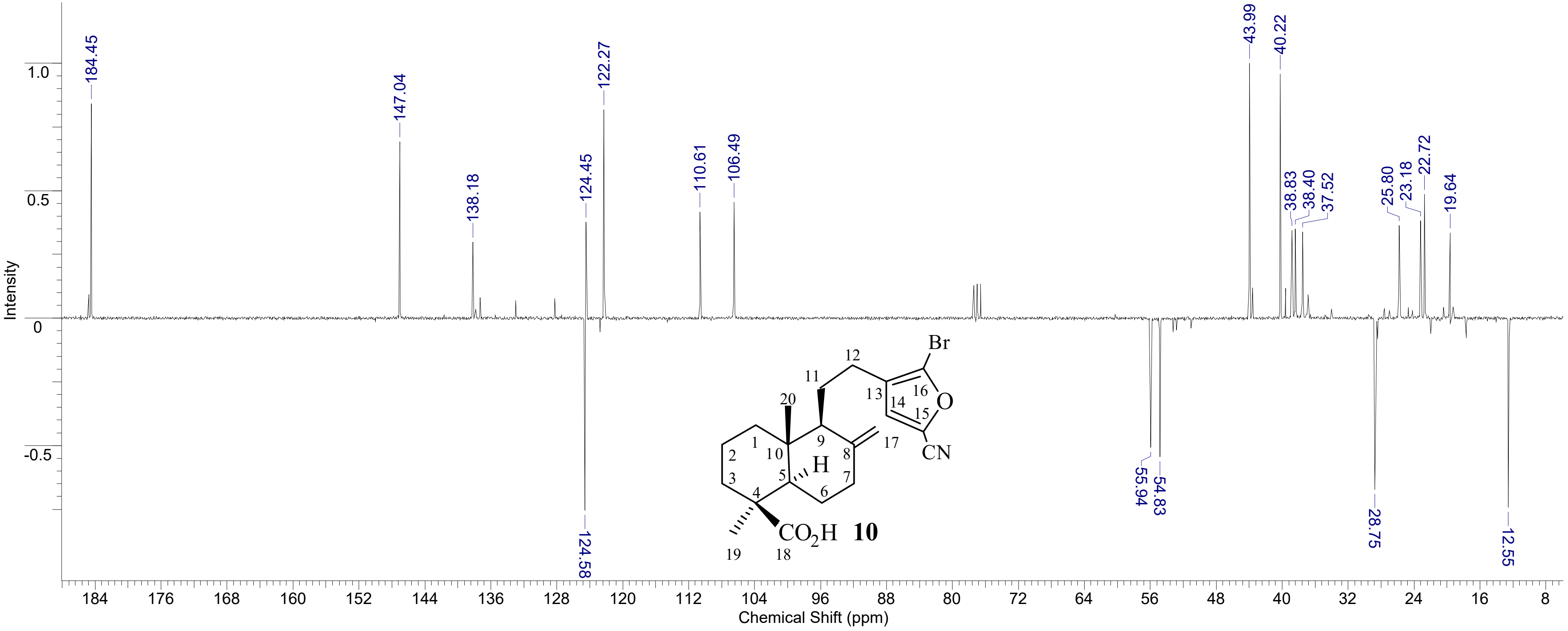

(1S,4aR,5S,8aR)-5-(2-(2-bromo-5-(hydroxymethyl)furan-3-yl)ethyl)-1,4a-dimethyl-6-methylenedecahydronaphthalene-1-carboxylic acid (11)

(^{1}H NMR, 300 MHz, $CDCl_3$)

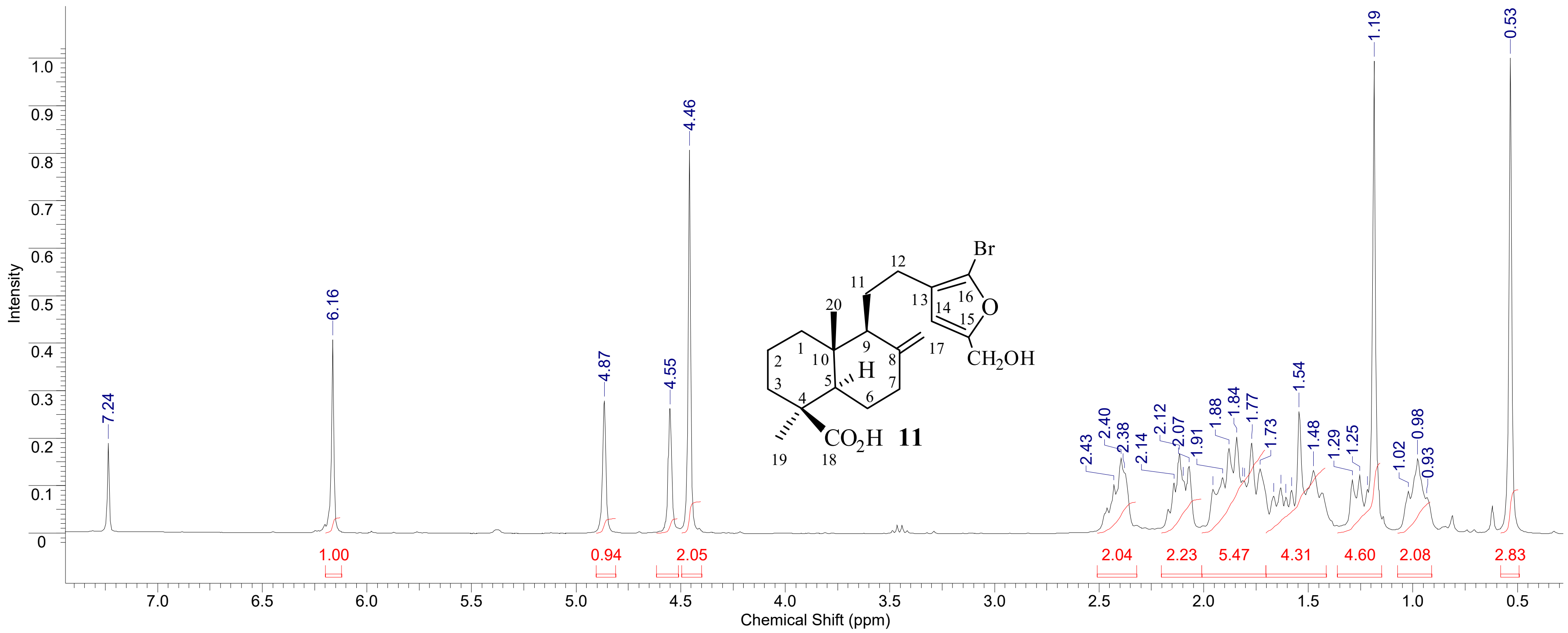

(1S,4aR,5S,8aR)-5-(2-(2-bromo-5-(hydroxymethyl)furan-3-yl)ethyl)-1,4a-dimethyl-6-methylenedecahydronaphthalene-1-carboxylic acid (11)

(^{13}C NMR, 126 MHz, $CDCl_3$)

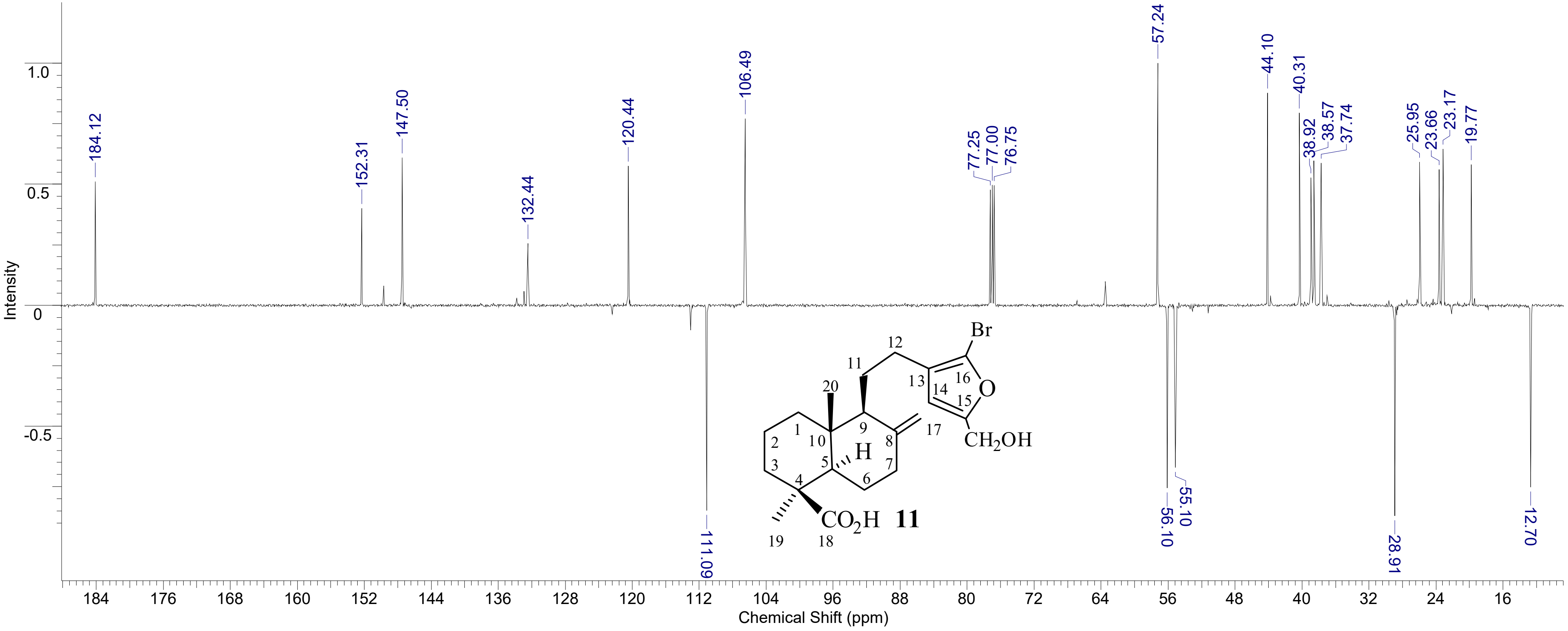

(1S,4aR,5S,8aR)-5-(2-(2-bromo-5-((methylamino)methyl)furan-3-yl)ethyl)-1,4a-dimethyl-6-methylenedecahydronaphthalene-1-carboxylic acid

(12) (^{1}H NMR, 500 MHz, $CDCl_3$)

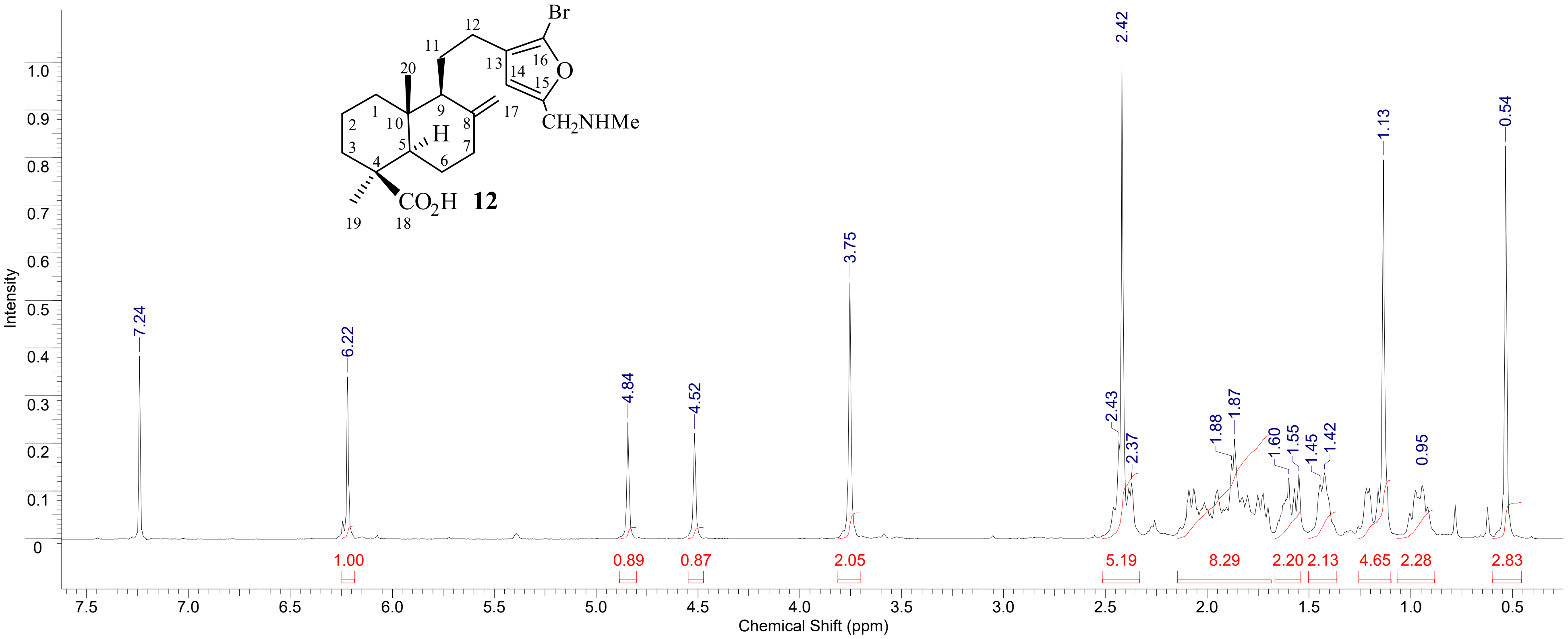

(1S,4aR,5S,8aR)-5-(2-(2-bromo-5-((methylamino)methyl)furan-3-yl)ethyl)-1,4a-dimethyl-6-methylenedecahydronaphthalene-1-carboxylic acid (12) (^{13}C NMR, 75 MHz, $CDCl_3$)

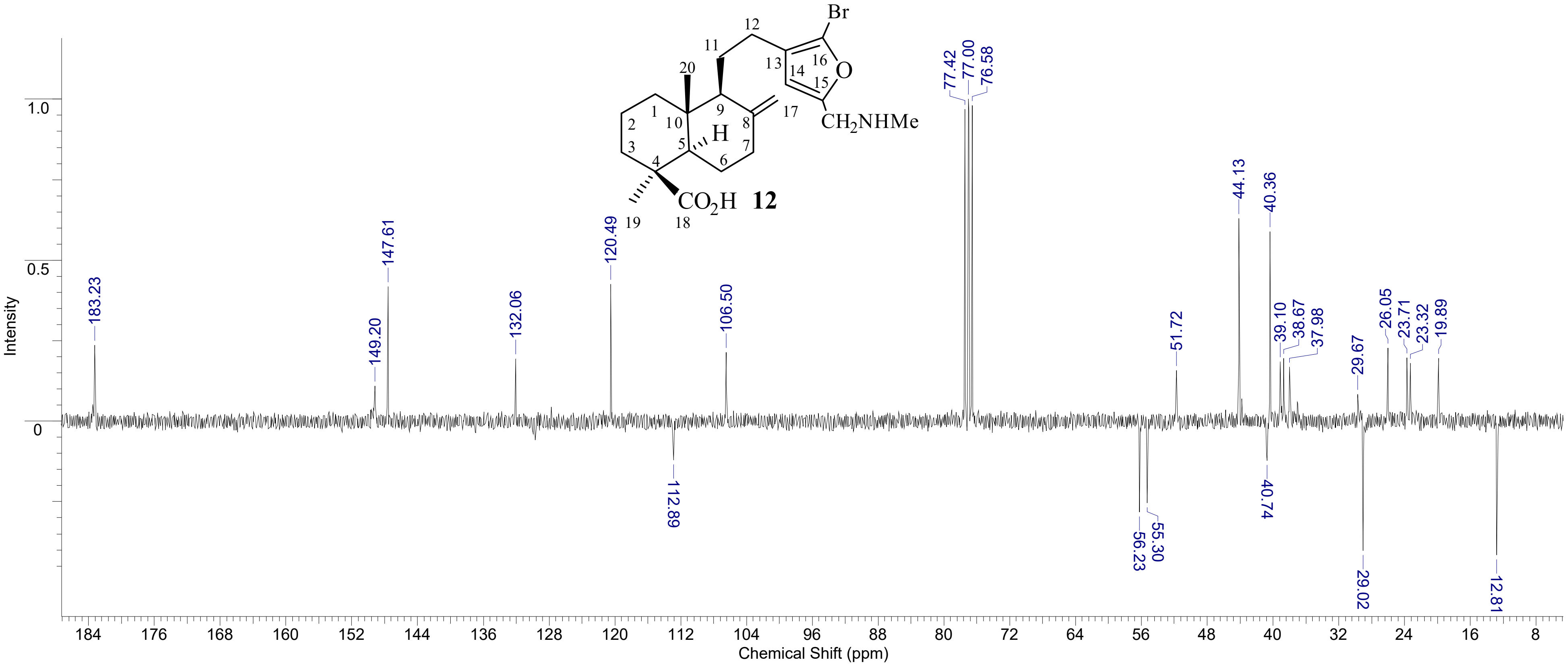

(1S,4aR,5S,8aR)-5-(2-(5-formyl-2-phenylfuran-3-yl)ethyl)-1,4a-dimethyl-6-methylenedecahydronaphthalene-1-carboxylic acid (*13a*)

(^{1}H NMR, 500 MHz, $CDCl_3$)

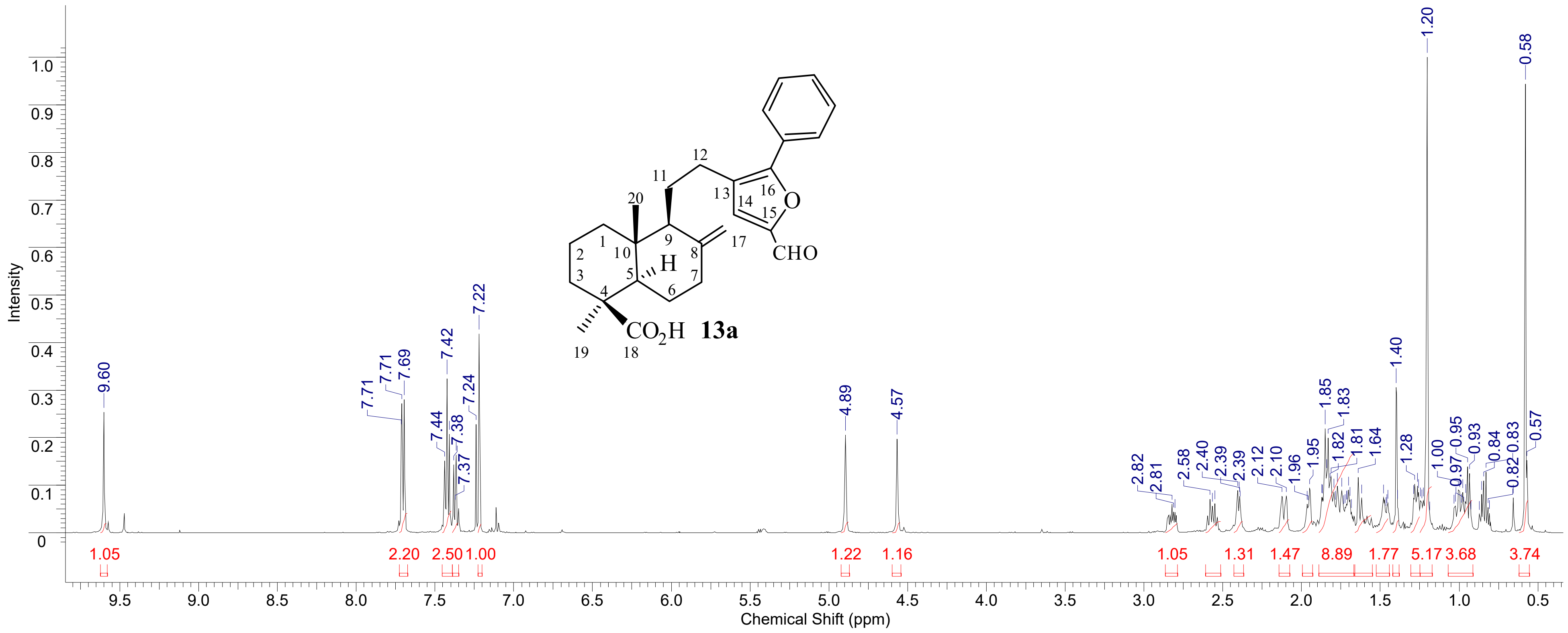

(1S,4aR,5S,8aR)-5-(2-(5-formyl-2-phenylfuran-3-yl)ethyl)-1,4a-dimethyl-6-methylenedecahydronaphthalene-1-carboxylic acid (**13a*****)***

(^{13}C NMR, 151 MHz, $CDCl_3$)

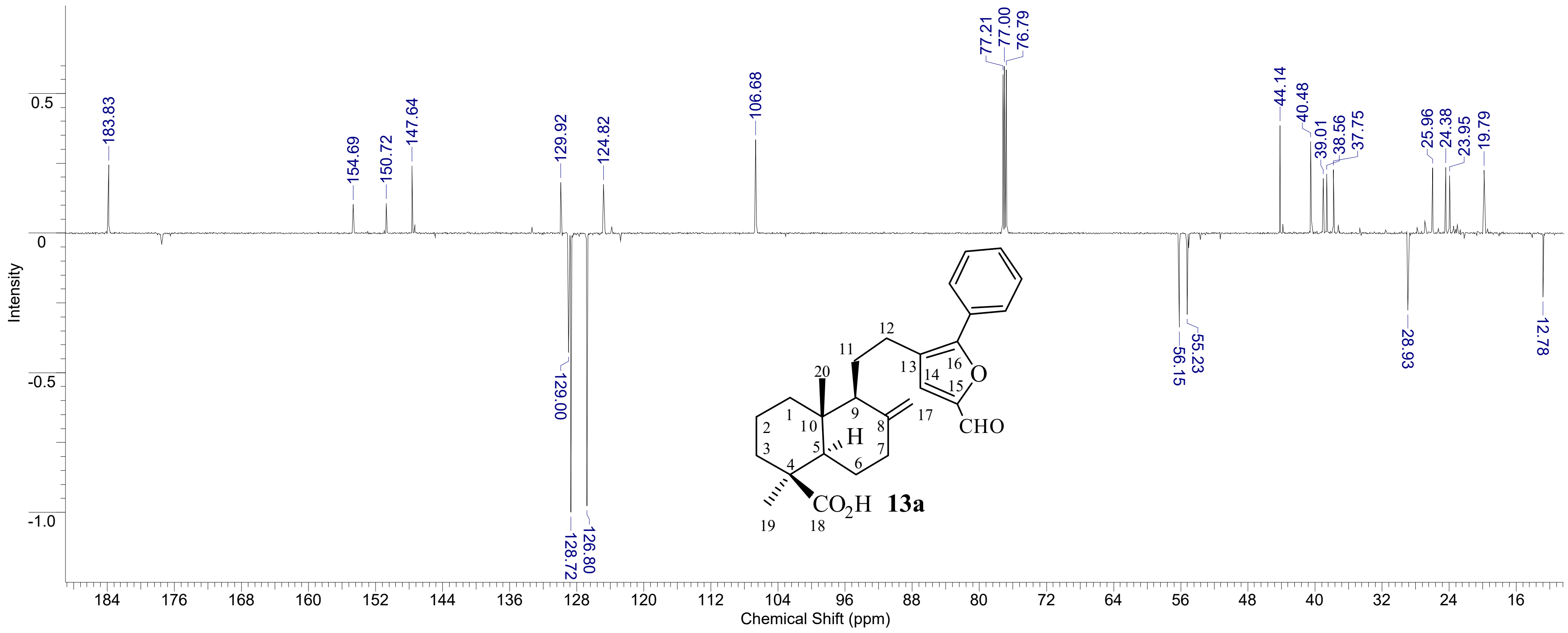

(1S,4aR,5S,8aR)-5-(2-(2-(4-cyanophenyl)-5-formylfuran-3-yl)ethyl)-1,4a-dimethyl-6-methylenedecahydronaphthalene-1-carboxylic acid (13b)

(^{1}H NMR, 300 MHz, $CDCl_3$)

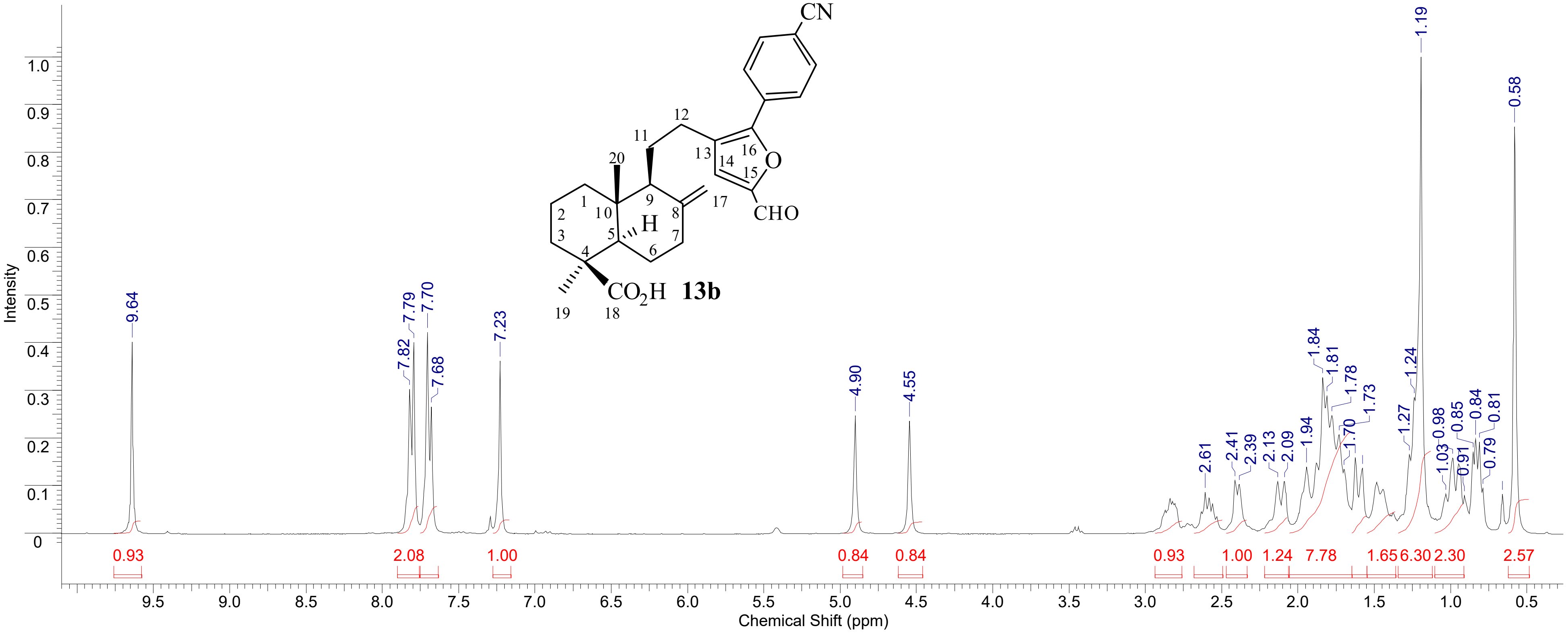

(1S,4aR,5S,8aR)-5-(2-(2-(4-cyanophenyl)-5-formylfuran-3-yl)ethyl)-1,4a-dimethyl-6-methylenedecahydronaphthalene-1-carboxylic acid (13b)

(^{13}C NMR, 101 MHz, $CDCl_3$)

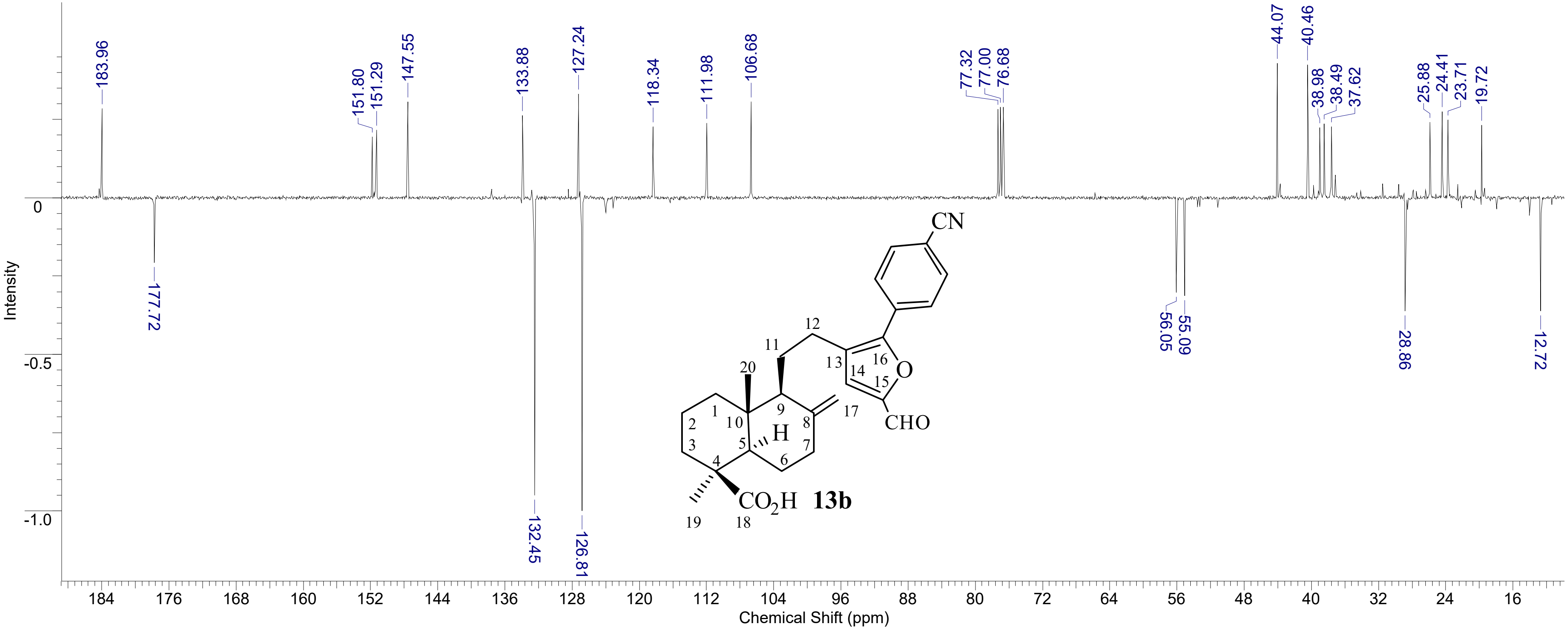

(1S,4aR,5S,8aR)-5-(2-(5-formyl-2-(3,4,5-trimethoxyphenyl)furan-3-yl)ethyl)-1,4a-dimethyl-6-methylenedecahydronaphthalene-1-carboxylic acid

(13c) (^{1}H NMR, 400 MHz, $CDCl_3$)

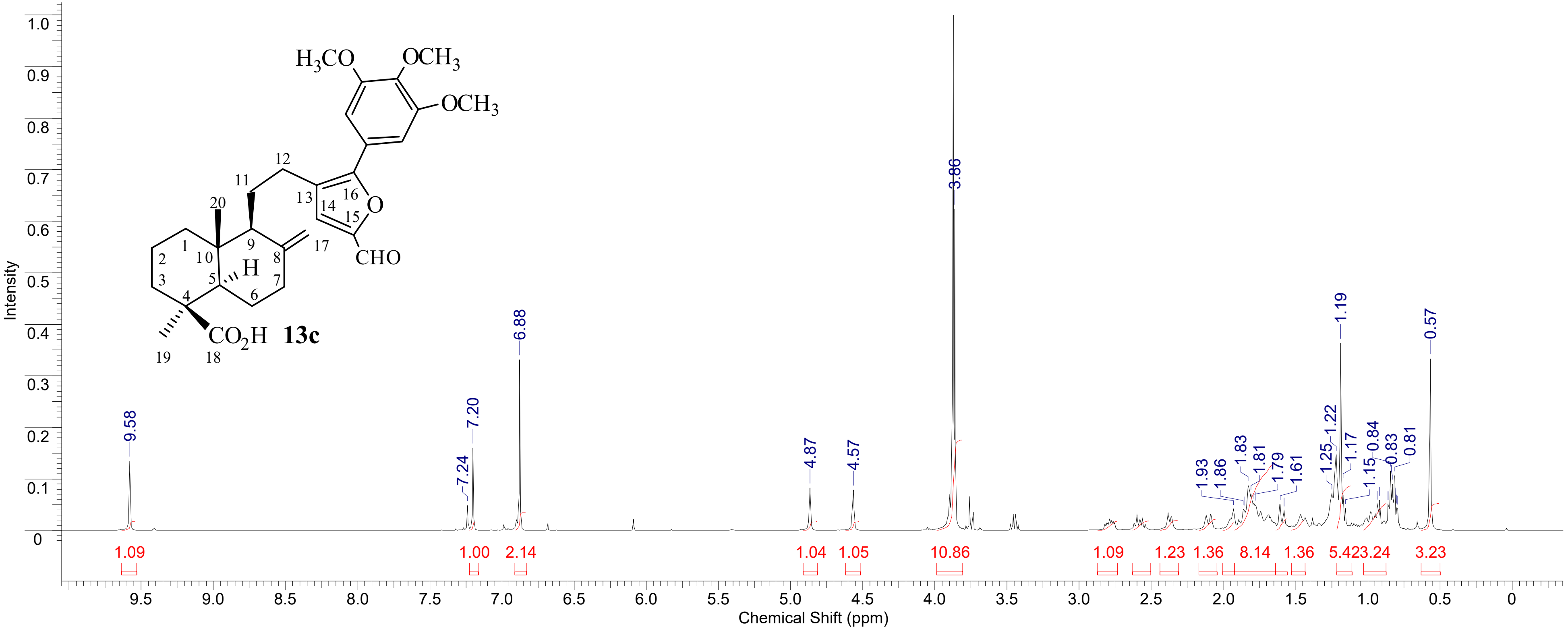

(1S,4aR,5S,8aR)-5-(2-(5-formyl-2-(3,4,5-trimethoxyphenyl)furan-3-yl)ethyl)-1,4a-dimethyl-6-methylenedecahydronaphthalene-1-carboxylic acid (13c) (^{13}C NMR, 126 MHz, $CDCl_3$)

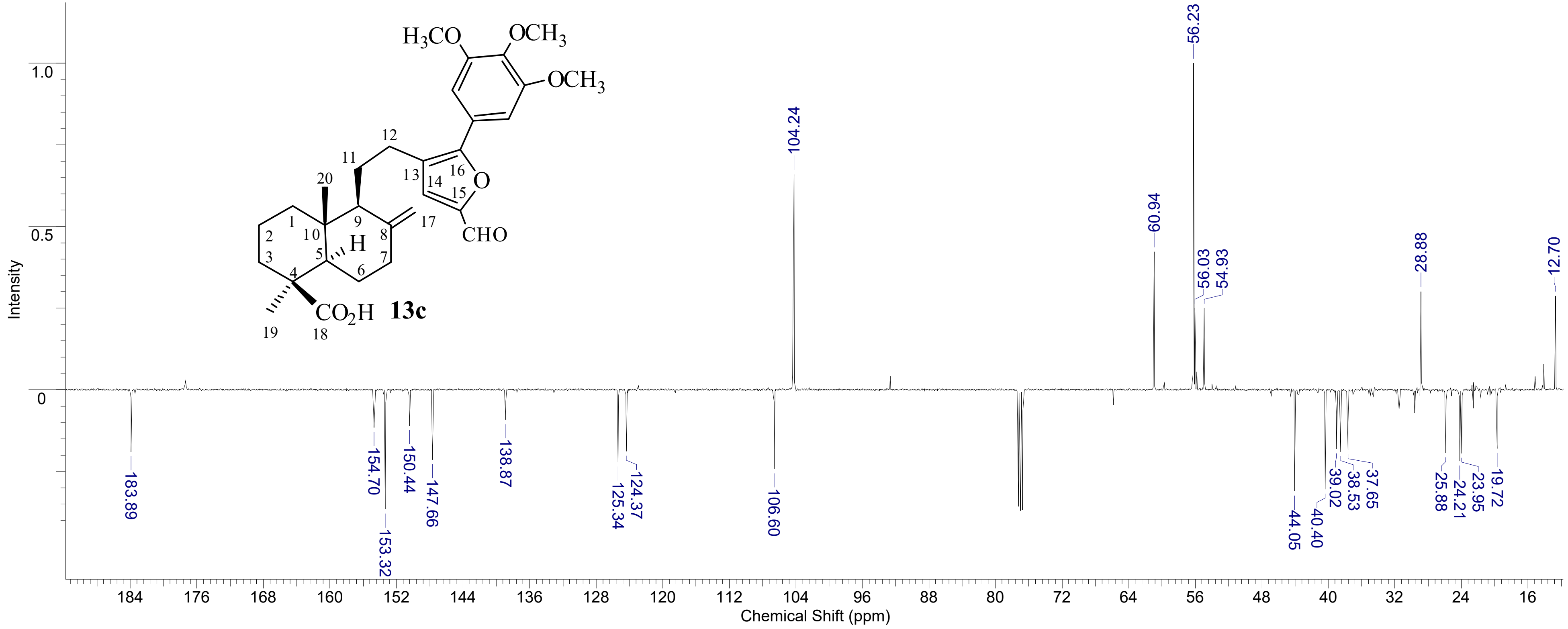

H3CO
OCH3
OCH3
CHO
CO2H
13c
Intensity
Chemical Shift (ppm)
183.89
154.70
153.32
150.44
147.66
138.87
125.34
124.37
106.60
104.24
60.94
56.23
56.03
54.93
44.05
40.40
39.02
38.53
37.65
28.88
25.88
24.21
23.95
19.72
12.70

(1S,4aR,5S,8aR)-5-(2-(5-cyano-2-phenylfuran-3-yl)ethyl)-1,4a-dimethyl-6-methylenedecahydronaphthalene-1-carboxylic acid (**14a*****)** (^{1}H NMR, 400 MHz, $CDCl_3$)

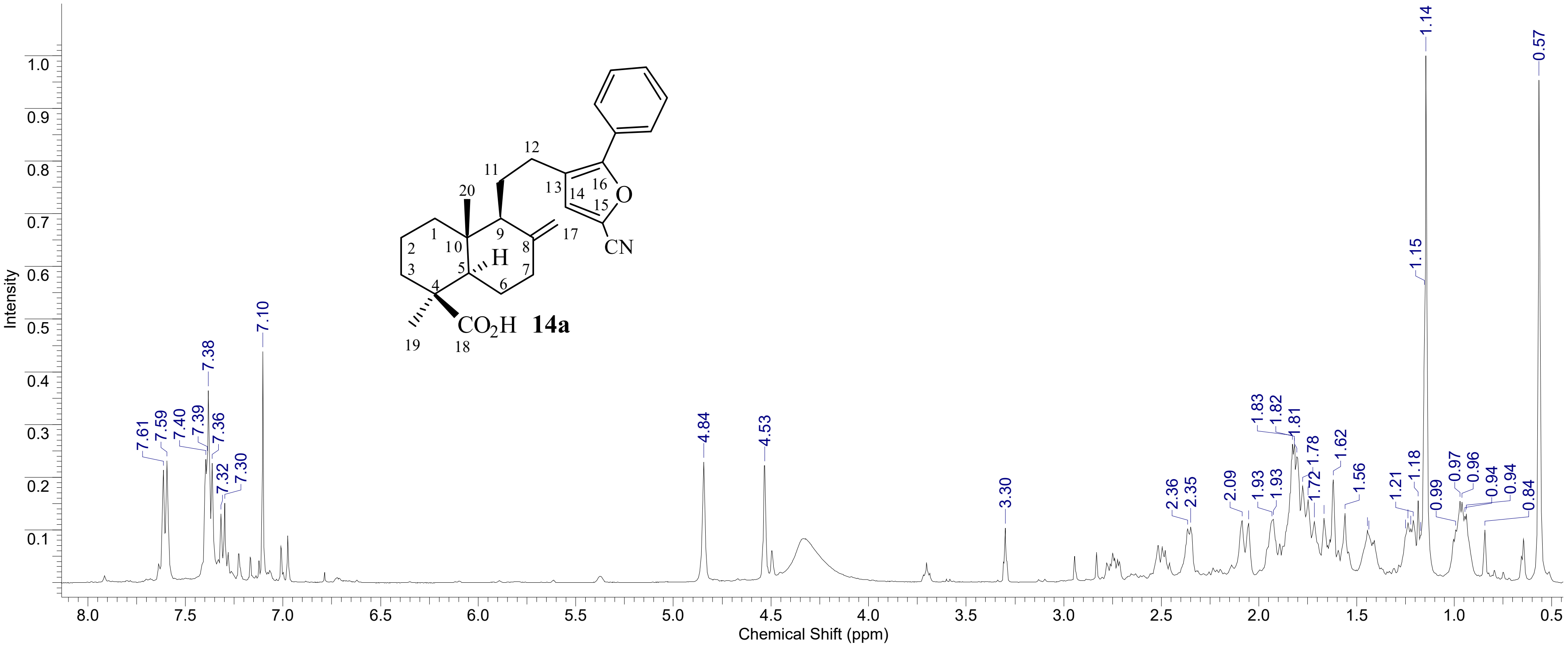

(1S,4aR,5S,8aR)-5-(2-(5-cyano-2-phenylfuran-3-yl)ethyl)-1,4a-dimethyl-6-methylenedecahydronaphthalene-1-carboxylic acid (*14a*)

(^{13}C NMR, 126 MHz, $CDCl_3$)

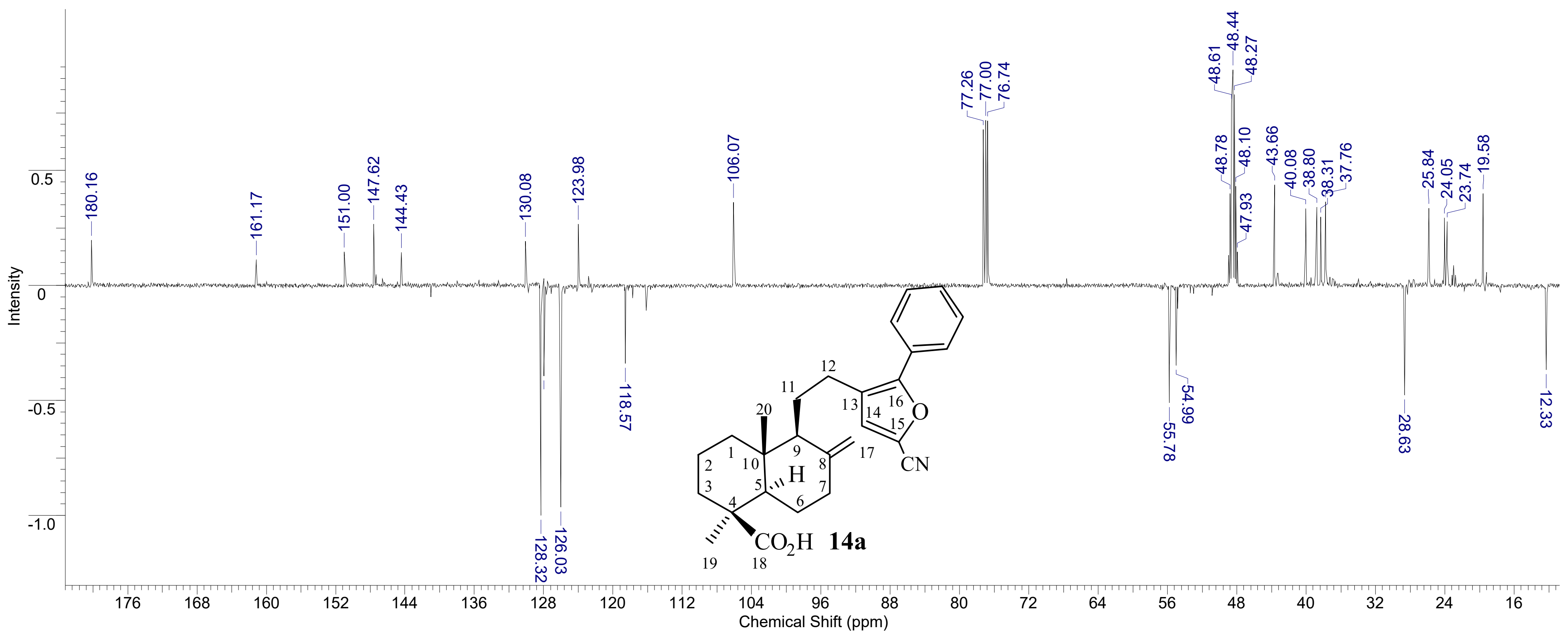

Supplement: Supplementary file 1 [file molecules-27-02643-s001.zip › molecules-1682495-supplementary.pdf]
